# The Role of Exercise in the Interplay between Myokines, Hepatokines, Osteokines, Adipokines, and Modulation of Inflammation for Energy Substrate Redistribution and Fat Mass Loss: A Review

**DOI:** 10.3390/nu12061899

**Published:** 2020-06-26

**Authors:** Adrian M. Gonzalez-Gil, Leticia Elizondo-Montemayor

**Affiliations:** 1 Tecnologico de Monterrey, Escuela de Medicina y Ciencias de la Salud, Ave. Morones Prieto 3000, Monterrey N.L. 64710, Mexico; amgonzalezgil@hotmail.com; 2 Tecnologico de Monterrey, Center for Research in Clinical Nutrition and Obesity, Ave. Morones Prieto 300, Monterrey N.L. 64710, Mexico; 3 Tecnologico de Monterrey, Cardiovascular and Metabolomics Research Group, Hospital Zambrano Hellion, San Pedro Garza Garcia P.C. 66278, Mexico

**Keywords:** exercise, physical activity, fat mass loss, obesity, energy substrate redistribution, myokines, hepatokines, osteokines, adipokines, inflammation

## Abstract

Exercise is an effective strategy for preventing and treating obesity and its related cardiometabolic disorders, resulting in significant loss of body fat mass, white adipose tissue browning, redistribution of energy substrates, optimization of global energy expenditure, enhancement of hypothalamic circuits that control appetite-satiety and energy expenditure, and decreased systemic inflammation and insulin resistance. Novel exercise-inducible soluble factors, including myokines, hepatokines, and osteokines, and immune cytokines and adipokines are hypothesized to play an important role in the body’s response to exercise. To our knowledge, no review has provided a comprehensive integrative overview of these novel molecular players and the mechanisms involved in the redistribution of metabolic fuel during and after exercise, the loss of weight and fat mass, and reduced inflammation. In this review, we explain the potential role of these exercise-inducible factors, namely myokines, such as irisin, IL-6, IL-15, METRNL, BAIBA, and myostatin, and hepatokines, in particular selenoprotein P, fetuin A, FGF21, ANGPTL4, and follistatin. We also describe the function of osteokines, specifically osteocalcin, and of adipokines such as leptin, adiponectin, and resistin. We also emphasize an integrative overview of the pleiotropic mechanisms, the metabolic pathways, and the inter-organ crosstalk involved in energy expenditure, fat mass loss, reduced inflammation, and healthy weight induced by exercise.

## 1. Introduction

Obesity, characterized by abdominal fat gain, is a complex multifactorial disease considered by many as a 21st century epidemic [1]. Its incidence is expected to continue given that the energy surplus results not only in increased body fat, specially abdominal fat, but also in numerous physiologic derangements and cardiometabolic complications and increased morbidity and mortality [1,2,3,4,5,6,7,8,9]. Currently considered a cornerstone in the management and prevention of obesity, exercise has been demonstrated to have a myriad of health benefits [9,10]. Of concern, less than 5% of adults from the U.S. follow the recommended guideline for physical activity of a minimum of 150 min per week [11].

Evidence suggests that M2 alternatively activated macrophage (Mφ) infiltrates are crucial for beige and brown adipose tissue (BAT) activity and that proinflammatory cytokines impair BAT function [12]. In patients with obesity, BAT function is significantly impaired, partially explained by the systemic proinflammatory state and the accompanying catecholamine-resistance that ensues [13], which is driven by proinflammatory M1 Mφ and CD4+ Th1-cell infiltrates in adipose tissue (AT) [14]. Although by far more studied in rodents than in humans [15], induction of beige adipocytes by exercise or functional enhancement of existing BAT has shown to improve total energy expenditure and insulin sensitivity, contributing to fat and weight loss [16,17,18,19]. Furthermore, the hallmark systemic low-grade proinflammatory state that accompanies obesity has been described to contribute in part to insulin resistance in skeletal muscle (SkM) and in the liver, which compromises their normal metabolic functions [20]. In SkM, insulin resistance has been shown to impair glucose uptake and glycogen storage due to decreased glucose transporter 4 (GLUT4) translocation, a state that is maintained by the ectopic accumulation of lipids (lipotoxicity) that results from increased circulating free fatty acids and their impaired catabolism. These latter events are driven by reduced expression and/or function of fatty acid transporters and enzymes involved in lipid oxidation, which results in further impairment of signaling downstream of the insulin receptor [21] by DAG-induced activation of PKC and phosphorylation of serine residues in IRS proteins [20]. Of interest, exercise is known to upregulate proteins and enzymes that participate in fatty acid oxidation and to increase insulin sensitivity and glucose uptake in SkM [21], as well as to promote a generalized anti-inflammatory state [9]. In the liver, hepatic insulin resistance, among other effects, has proved to result in increased gluconeogenesis, lipogenesis, and synthesis of very-low-density lipoproteins (VLDL) [22] that carry cholesterol and triglycerides for their accretion in peripheral tissues; if this accretion is deregulated, it may lead to an atherogenic lipid profile, increased fat mass, and lipotoxicity. Additionally, if the capacity of the liver to synthesize and export lipids is overrun by excess substrate, intrahepatic lipid accumulation and steatosis eventually ensue, which have been described to exacerbate not only local, but also systemic insulin-resistance [23]. Interestingly, exercise is known to decrease hepatic steatosis [24]. Finally, obesity has also been associated with altered central regulation of energy expenditure and appetite, and it is generally agreed that exercise can positively influence central energy balance in the long term [25]. Altogether, considering the pleiotropic beneficial metabolic effects of exercise, the search for the mechanisms through which it favors fat mass loss and metabolic health has been an ongoing focus of interest. Of these, novel circulating molecules induced by exercise, such as myokines, hepatokines, osteokines, immune cytokines, and adipokines regulate metabolic pathways and inter-tissue crosstalk by means of autocrine, paracrine, and endocrine effects [2], and could thus be at least partially responsible for preventing, or even reverting, obesity and the obesity-associated metabolic dysfunction in target organs.

Myokines such as irisin, interleukin-6 (IL-6), interleukin-15 (IL-15), meteorin-like (METRNL), and β-aminoisobutyric acid (BAIBA) have been consistently shown to be released by SkM in response to exercise. These exert beneficial physiologic and metabolic effects not only in SkM itself but also in distant tissues such as white adipose tissue (WAT), bone, liver, the central nervous system (CNS), and cells of the immune system to drive a systemic anti-inflammatory and insulin-sensitive state, permissive for optimization of total-body energy expenditure [5,7,26,27]. Likewise, hepatokines such as FGF21, ANGPTL4, and follistatin, released during and after exercise, may influence WAT and SkM metabolism to favor redistribution of lipid-derived metabolic fuel towards catabolic pathways [4]. On the other hand, the detrimental hepatokines fetuin A and selenoprotein P, which are increased in insulin-resistant states [23], could blunt the beneficial effects of exercise-inducible factors. The decrease of such unfavorable hepatokines and the reversal of the hepatic steatosis that frequently accompanies the obese state [3,23] could be achieved with exercise interventions. Moreover, the osteokine osteocalcin (OCN) is released by bone following bouts of exercise and cooperates with myokines to increase FFA utilization in multiple tissues [28]. The combined actions of these factors, either directly or indirectly, likely result in decreased triglyceride (TG) accretion in visceral fat depots and in increased mobilization and fatty acid oxidation (FAO) in the liver and SkM. These changes further increase available SkM bulk for glucose disposal, decreasing lipogenesis in both the liver and WAT and decreasing inflammatory mediators that result in insulin resistance, BAT dysfunction, and dysregulation of the hypothalamic neurons controlling energy balance. Furthermore, exercise-induced loss of visceral adipose tissue (VAT) could reestablish normal levels of the anorexigenic adipokine leptin, increase levels of the insulin-sensitizing and anti-inflammatory adipokine adiponectin, and decrease the levels of proinflammatory immune cytokines in general [6,9], thus promoting healthy weight maintenance and metabolic health. Further adding a level of complexity, exercise-inducible factors engage in intricate cross-regulation with one another and with the cytokines that determine inflammatory status, thus engaging in synergistic crosstalk while mitigating metabolic stress in target organs.

Literature on this topic is vast and has been reviewed by others but on a separate basis, by describing either individual molecules or groups of factors, proteins, hormones, or molecules [2,3,4,5,6,7,8,9,29]. However, to our knowledge, no review has yet provided a comprehensive integrative overview of these mostly novel molecular players. Hence, the aim of this review is to provide a state-of-the-art holistic view of the mechanisms through which exercise induces abdominal fat loss and maintains metabolic homeostasis and a healthy weight. Given the broad nature of this topic, for which we conducted an exhaustive literature search in the PubMed database, we sought to summarize the existing evidence in the form of a narrative review. We first describe the individual myokines, hepatokines, cytokines, osteokines, and adipokines that have been shown to be modified in response to exercise in adults and that, through either direct or indirect mechanisms, contribute to fat loss and weight loss. We then include both clinical and preclinical studies to highlight the possible mechanisms of these exercise-inducible factors. Finally, we integrate this information and propose a model of inter-organ crosstalk in which these molecules and their metabolic pathways interplay, through which exercise mediates its beneficial effects on fat loss, weight maintenance, and healthy metabolism. We also comprise a set of figures showing the intertwining pathways.

## 2. Redistribution of Energy Substrates in Response to Acute and Chronic Exercise

An acute bout of exercise brings about the activation of multiple neuroendocrine pathways that result in short-term and long-term systemic metabolic adaptations. In the short term, breakdown of ATP and phosphocreatine, leading to anaerobic glycolysis in the exercising SkM, has been described [30]. Increased metabolic demands are accomplished through mechanisms that are mostly mediated by the sympathetic nervous system (SNS), such as lipolysis in WAT dependent on the activation of hormone sensitive lipase (HSL) by catecholamines [18,30], mobilization of hepatic glycogen stores, and antagonism of the anabolic actions of insulin through the increased release of cortisol and growth hormone [31] that favors TG catabolism in WAT [32]. The net result is increased available glucose and free fatty acids (FFAs) for the exercising SkM [30].

With chronic aerobic exercise (e.g., daily training), the cellular mechanisms involved in oxidative metabolism are upregulated in SkM in a time-dependent manner but are immediately reverted to baseline following abstinence from exercise [30]. Indeed, SkM undergoes multiple adaptive mechanisms, including increased mitochondrial biogenesis, expression of fatty acid transporters, activity of oxidative enzymes and of those involved in the electron transport chain in the mitochondria, and, importantly, SkM hypertrophy [30]. Additionally, continued FAO takes place during the post-exercise early recovery period (i.e., 4 h) and continues at a lesser degree (still above baseline) for as long as 24 h [32]. The basal metabolic rate (BMR) increases 5–10% from baseline after exercise, but, intriguingly, this effect has been reported to last up to 48 h post-exercise [33]. Therefore, mechanisms other than continued FAO must also account for the increased BMR observed during the post-exercise period. Increased total energy expenditure could result from a reestablished hypothalamic setpoint in control of energy expenditure, increased excess nutrient disposal, and thermogenesis by BAT [34]. Metabolic energy demands may also be met through redistribution of energy substrates due to increased SkM mass with oxidative metabolic adaptations that allow for enhanced consumption of glucose or glycogen storage in the resting state. This, together with the potent insulin-sensitizing effect of exercise [35], limits the amount of substrate available for lipogenesis in WAT and in the liver. In the latter, TGs are transported into the circulation and eventually accreted in WAT [36]. Indeed, considering that SkM represents approximately half of the total body mass, that it accounts for nearly 80% of insulin-stimulated glucose uptake, and that it is a major determinant of the basal metabolic rate, even small increases in the energy demand in SkM can have significant effects on total-body energy expenditure [37], even in the basal state. Thus, exercise can favor the redistribution of energetic substrates to SkM, where they are expended, instead of to WAT, where they are stored, thus maintaining physiologic levels of fat mass. As discussed later, exercise factors may be involved in many of these processes and may therefore influence fat mass. Regardless of the mechanism, the post-exercise period appears to be crucial for the observed VAT loss that accompanies exercise.

The mechanisms through which “exercise-inducible factors” may have a global effect on metabolism by influencing any of the processes outlined above are described in the following sections and summarized in Tables 1–4. Some of these factors are not exclusively synthesized by a specific type of tissue. Hence, they are classified based on their most important source following bouts of exercise.

## 3. The Role of Myokines in Metabolic Pathways during Exercise

Myokines are a broad group of muscle-secreted proteins, peptides, and metabolites that have been reported to hold endocrine activity, most of which are secreted in response to SkM contraction, bouts of physical activity, and other endocrine signals. Well-studied myokines [38] are summarized in Table 1, where brief overview on their physiologic role, regulation, and changes in levels or activity in response to exercise is provided.

### 3.1. Irisin

Irisin is released primarily from SkM after physical exercise, and to a lesser extent by WAT, from its precursor fibronectin type III domain-containing protein 5 (FNDC5), its cleavage probably involving ADAM family proteases [120]. When originally described, it was found to exert browning of WAT, increase energy expenditure, improve insulin sensitivity, and induce weight loss, through upregulation of UCP1 in exercised mice overexpressing PGC-1α [57]. Irisin concentrations have been found to be increased in obesity in humans [39]. Irisin was described to stimulate lipolysis via HSL and to inhibit lipid accumulation in adipocytes [58,64]. In SkM, after physical activity, irisin induced downstream activation of glucose and FFA uptake and oxidation [62]. It has favored not only M2 Mφ polarization, but also reversion of an already established M1-polarized proinflammatory state [59]. Irisin induces VAT loss as well. After an 8-week endurance training in healthy adults, increased circulating irisin levels were positively correlated with the amount of visceral fat loss measured by DEXA scan [121]. The anti-inflammatory effect of irisin in VAT might potentiate its browning properties while sensitizing white adipocytes in situ to the action of catecholamines, further contributing to a decrease in VAT mass. Released FFAs from VAT could then be redistributed to metabolically active tissues, given that irisin directly promotes expression of genes involved in FFA uptake and oxidation and stimulates hypertrophy [44], thus increasing available SkM mass for FAO and glucose disposal, which further decreases available substrates for lipogenesis. Moreover, the antioxidative and anti-inflammatory effects of irisin have also been described in hepatocytes, which may help decrease hepatic steatosis [65] and favor a healthy hepatokine profile which also contributes to decreased fat mass [23]. Although still controversial [56], it is generally agreed that irisin levels increase transiently following acute bouts of aerobic and resistance exercise [40], which could contribute to loss of fat mass. Data on chronic exercise are less conclusive. A meta-analysis has described a decrease in irisin levels with resistance training and a tendency to decrease with endurance training [41]. These results could be attributed to decreased metabolic stress and inflammation that follows exercise-associated fat loss, rather than as a consequence of exercise per se, and to the inclusion of studies that did not measure irisin levels immediately following exercise bouts, considering its proposed short life in circulation [40].

### 3.2. IL-6

Increased concentration of IL-6, secreted by adipocytes and monocytes and activated through the TLR4-NF-κB pathway, has been classically associated with proinflammatory responses and insulin resistance in obesity. However, when released by SkM during exercise bouts and secreted in response to activation of the MAPK/JNK/AP-1 pathway, IL-6 has been shown to exert an anti-inflammatory and insulin-sensitizing action [122]. IL-6 serum levels have been shown to increase following bouts of aerobic exercise, with exercise duration as the main determinant of the increase (e.g., 5-fold following 30 min of running at 75% maximal oxygen consumption (VO_2_ max) to 100-fold during marathon running) [8,70]. Obese adults receiving tocilizumab, subjected to 12 weeks of endurance exercise, showed a blunted exercise-induced loss of visceral fat mass compared with controls, as measured by DEXA [78]. Thus, IL-6 appears to be crucial in exercise-induced loss of VAT. It has been shown to directly increase GLP-1 in mice [73] and to stimulate SNS, increasing WAT lipolysis and “browning” and BAT thermogenesis [19]. By directly promoting lipolysis and mediating metabolic adaptation to exercise in SkM [75], IL-6 favors glucose disposal, which ultimately results in SkM effectively utilizing FFAs derived from VAT lipolysis and decreased available lipogenic substrate.

### 3.3. IL-15

IL-15 is produced by SkM in response to exercise [123], acting by autocrine means. IL-15 augments glucose uptake in SkM by increasing transcription [124] and membrane translocation of GLUT4 via JAK3/STAT3 signaling [125], and augments PPARδ and PGC-1α activity, favoring mitochondrial biogenesis and FAO [84,91,126]. It decreases fat mass by promoting influx of energetic substrates to SkM, which limits FFA deposits in VAT and in adipocytes [127]. The global effect of IL-15 on fat mass appears to be significant, as exemplified in a murine model overexpressing IL-15 in which the percentage of visceral fat mass, measured by DEXA, was approximately half when compared with controls [88]. Most of the studies in both healthy and obese adults have found increased circulating IL-15 immediately following acute bouts of aerobic exercise (e.g., running or cycling at 55–75% of age-predicted maximum heart rate for 30–120 min) [128,129,130]. IL-15 concentrations in SkM biopsies are markedly increased despite no apparent increase in the circulation following exercise [85]. IL-15 promotes SkM hypertrophy, facilitating utilization of FFAs that would otherwise be reincorporated to VAT. In obese/T2DM subjects, soluble IL-15Rα is released in greater amounts, impairing upregulation of SkM oxidative metabolism or lipolysis in WAT [84].

### 3.4. Meteorin-Like (METRNL)

In SkM, METRNL is upregulated by activation of the PGC-1α4 isoform. It has been shown to induce PPARγ activity in WAT to increase preadipocyte differentiation and insulin sensitivity [102]. It was found to promote M2 Mφ polarization [98], enhance oxidative metabolism in SkM and WAT “browning” [100], stimulate FAO, and suppress FFA-induced inflammation and insulin resistance in SkM via activation of AMPK and PPARδ. A recent study found a positive correlation between METRNL and irisin levels in patients with T2DM [92]. Regulation of METRNL by exercise in humans has been scarcely evaluated. Increased METRNL gene expression at 1 and 4 h following an acute bout of combined resistance and endurance exercise in healthy young adult males was described [98]. METRNL mRNA expression was also found upregulated in SkM biopsies taken 3 h into the recovery period compared with the immediate post-exercise period both before and after 20 days of standardized high-intensity interval training (HIIT; 5 × 4-min bouts at 80% pre-training peak power output) in healthy young males [97]. A recent study identified a significant inverse association between visceral fat area, evaluated with DEXA, and circulating levels of METRNL in adults with T2DM [131], advocating its possible involvement in fat mass regulation. Overall, the scant evidence suggests that METRNL may play a role in exercise-induced loss of fat mass. It could also initiate a cascade of myokines that synergistically increase energy expenditure, through increased glucose and FFA disposal, and thermogenesis in BAT, possibly attenuating insulin resistance, which indirectly results in loss of fat mass.

### 3.5. β-Aminoisobutyric Acid (BAIBA)

β-Aminoisobutyric acid (BAIBA), released from contracting SkM, was originally identified in culture supernatant of murine skeletal myocytes and in the circulation of both chronically exercised mice and those overexpressing PGC-1α, promoting WAT “browning” and increasing hepatic β-oxidation through PPARα-dependent mechanisms. Fat mass loss associated with increased total energy expenditure was also observed [104]. BAIBA promoted reversal of weight gain and adiposity in HFD-induced severely obese mice [114], attributed to restored hypothalamic neuronal function and possibly sensitivity to anorexigenic hormones. BAIBA promotes increased VAT lipolysis, increased FFA oxidation, and glucose uptake in SkM [110,111,112]. In the liver, a simultaneous decrease in de novo lipogenesis and increased FAO [112,113] could also reduce accretion of diet-derived TGs and excess carbohydrates in VAT. However, BAIBA remains understudied in humans. A study showed negative correlations between BAIBA levels and percentage of body fat, total fat mass, subcutaneous adipose tissue (scAT), and VAT, and a positive association with lean mass in obese vs. healthy adults [105]. Regarding exercise interventions, two studies have reported increased circulating levels of BAIBA following aerobic exercise, e.g., cycling for 1 h at 40% maximal power output [106] or after 20 weeks of 30–50 min cycling sessions at 55–75% VO_2_ max [104] in healthy young men. A trend toward a negative correlation between BAIBA and BMI [104] was shown, while another study reported no increase [107]. Thus, the relationship between exercise-induced BAIBA and anthropometric markers warrants further study.

### 3.6. Myostatin

Myostatin was the first identified muscle-secreted factor and is known to promote SkM atrophy [115]. It is the only myokine known to be downregulated by acute and chronic endurance- and resistance-type exercise in both rodents and humans [115]. Its expression is increased in human obesity and strongly associated with insulin resistance by downregulating GLUT4 expression and by decreasing phosphorylation of IRS1 [26]. On the contrary, inhibition of myostatin upregulates oxidative metabolism in SkM [115]. Its absence in Mstn^-/-^ mice results in increased gene expression of enzymes and transcription factors involved in lipolysis, mitochondrial FAO, and WAT browning, as well as decreased VAT mass [132]. It contributes to the induction of PGC-1α, FNDC5, and irisin in SkM [27,49,133]. The possibility of an inter-myokine axis with irisin [44,49] and crosstalk with follistatin could represent a mechanism through which myostatin is decreased by exercise. In brief, exercise-induced inhibition of myostatin promotes SkM hypertrophy and oxidative metabolism, VAT lipolysis, and WAT browning, which may redistribute FFAs from lipogenesis to their utilization as metabolic fuel.

## 4. The Role of Hepatokines in Metabolic Pathways during Exercise

Hepatokines are novel hormone-like factors released from the liver. Clinical studies have suggested that exercise modulates the levels and the activity of FGF21, follistatin, ANGPTL4, and fetuin A, whose combined effect could decrease fat mass levels, as summarized in Table 2. In addition, selenoprotein P, although not directly modulated by exercise, may blunt the SkM adaptive response to exercise. Additionally, hepatokines may modulate the actions of other exercise-inducible factors, thus inhibiting fat accumulation; this favors SkM adaptation to exercise and an anti-inflammatory pro-browning environment that promotes loss of fat mass, attenuation of hepatic steatosis, and decreased insulin resistance.

### 4.1. Selenoprotein P

Excessive levels of selenoprotein P (SeP) have been shown in humans with metabolic diseases and are positively correlated with insulin resistance, which is consistent with its insulin-desensitizing effects observed in animal and in vitro models [23]. While its levels appear not to be regulated by exercise, perhaps due to insufficient exercise intensity or duration [4,50,134], SeP has been implicated in the “exercise resistance” phenomenon, which postulates that not all individuals derive the same beneficial effects from exercise. In animal models, obese SeP-deficient mice presented increased aerobic exercise capacity concomitant with enhanced mitochondrial biogenesis and function in SkM, an effect linked to increased ROS and activation of AMPK and to increased activity of PGC-1α [50]. In humans, high pre-training levels of SeP were predictive of decreased aerobic exercise capacity, measured as VO_2_ max [50], suggesting that FFAs released by VAT lipolysis during and after exercise are less efficiently utilized by SkM and are instead preferentially reincorporated into VAT via VLDL. A potential decrease in irisin, METRNL, and BAIBA further blunts the exercise-induced loss of fat mass. These mechanisms could partially explain why obese individuals, who have higher basal levels of SeP, appear to require a longer duration of exercise for efficient weight reduction compared with healthy controls [186].

### 4.2. Fetuin A

Fetuin A engages TLR-4 in adipocytes and Mφ to promote proinflammatory activation, in conjunction with FFAs [187,188]. Fetuin A has been shown to directly induce insulin resistance [189], suppress production of adiponectin by adipocytes [148], and induce pancreatic β-cell toxicity [152]. Notably, its levels have been found increased in obesity, especially associated with NAFLD [137], and are predictive for the development of T2DM [190]. Concordantly, increasing levels of fetuin A were associated with increasing VAT mass in elderly adults over a 5-year follow-up [191]. Regarding exercise, a recent meta-analysis found decreased fetuin A levels following completion of aerobic exercise regimes of varying frequency and duration (e.g., 1–12 weeks, 3–5 sessions per week of 40–70 min each) and intensity (e.g., 60–85% maximum heart rate), except in patients with T2DM, suggesting that modulation of fetuin A by exercise partially depends on the pre-existing metabolic status [138]. Another 12-week aerobic exercise training intervention, consisting of treadmill walking at 85% maximum heart rate for 60 min per session, decreased fetuin A levels in obese, insulin-resistant subjects, leading to decreased hepatic insulin resistance, total body fat, and fasting insulin levels [139]. Thus, decreased fetuin A levels induced by exercise could reduce VAT directly by decreasing FFA availability for uptake and TG accretion by WAT, while reducing VAT indirectly by reducing insulin resistance. Furthermore, decreased fetuin A may counteract VAT accumulation in part due to decreased activation of resident proinflammatory M1 Mφ that impair catecholamine-induced lipolysis and BAT function.

### 4.3. Fibroblast Growth Factor 21 (FGF21)

Exercise intensity is a determinant of the magnitude of FGF21 release from the liver; its levels have been found to be significantly higher in the recovery period than immediately post-exercise. Higher FGF21 levels were observed 1 h following treadmill running for 30 min at 80% VO_2_ max versus 50% VO_2_ max in healthy sedentary adults [155]. In the liver, FGF21 upregulates β-oxidation [160] and downregulates lipogenesis [167]. In WAT, it induces lipolysis [166] and browning [43], likely via upregulation of PGC-1α [192]. In the CNS, FGF21 stimulates the SNS, increasing BAT activity. Weight loss likely occurs [163,165] at the expense of VAT, as evidenced by a 15% mean decrease in abdominal fat content, measured by DEXA, in obese monkeys [165]. Interestingly, a recent study suggests that FGF21 may be involved in regulating preference for specific foods; specifically, it acts to reduce craving for sugary foods [168]. In summary, exercise-induced FGF21 could promote visceral fat mass loss by increasing lipolysis and thermogenesis in WAT (thereby redistributing energetic substrate to SkM), by decreasing sugary food intake (limiting de novo lipogenesis in the liver), and by reducing VLDL-mediated fat accretion in WAT. Paradoxically, FGF21 levels increase in patients with obesity [153], which might represent a state of FGF21 resistance [154]. Furthermore, patients with obesity-related insulin resistance have an impaired FGF21 upregulation after exercise [156,157]. Thus, increased frequency or duration of exercise may be needed to restore physiological regulation of FGF21 and thus its ability to decrease adiposity in obese subjects.

### 4.4. Angiopoietin-Like 4 (ANGPTL4)

Angiopoietin-like 4 (ANGPTL4) secretion by the liver has been primarily regulated during exercise bouts, following 3 h of ergometer cycling at 50% VO_2_ max, in response to increased glucagon-to-insulin ratio [170], and therefore its endocrine role is hypothesized [3]. In WAT, it was shown to stimulate lipolysis via induction of adipose triglyceride lipase [176,193] and to decrease the activity of LPL [178,194]. It has also been shown to inhibit pancreatic lipase, decreasing dietary fat absorption [179]. Anptl4**^-/-^** mice presented higher levels of visceral fat mass, visceral LPL activity, and WAT inflammation [195], highlighting the role of ANGPTL4 in the redistribution of lipoprotein-derived FFAs [4]. Although it is upregulated by SkM after aerobic or resistance exercise [171,172,196], surprisingly, an even greater degree of ANGPTL4 mRNA expression was found in the portion of SkM that remained inactive during the bout of exercise compared with the active SkM [172,196], representing a mechanism to redistribute FFA delivery from unexercised to exercised SkM. An autocrine role has also been proposed, given that ANGPTL4 is necessary for PPAR-δ-dependent expression of HSL [176]. In exercised SkM, oxidative capacity is increased, but in unexercised SkM, protection against lipotoxicity is possible. ANGPTL4 could selectively inhibit LPL in WAT to redirect FFAs for catabolism in SkM, rather than to their storage in WAT [197]. In summary, in the context of exercise, ANGPTL4 could redistribute FFA-derived metabolic fuel to SkM away from WAT, thus potentially inhibiting lipid accumulation in VAT.

### 4.5. Follistatin

In humans, circulating follistatin is primarily derived from the liver and, similar to FGF21 and ANGPTL4, increases in response to an augmented glucagon-to-insulin ratio in conditions such as fasting and exercise, including HIIT, continuous aerobic, and resistance modalities [182,198]. Studies in humans have identified follistatin as an exercise-inducible hepatokine that increases transitorily in the recovery period after a bout of exercise [117,134,183]. Expectedly, like FGF21, patients with obesity-related insulin resistance present increased basal levels of follistatin [157,180], but its exercise-induced release by the liver is blunted [157]. Follistatin might enhance SkM hypertrophy due to its ability to bind and neutralize myostatin [184,199], increasing the mass of SkM available for glucose and FFA uptake and oxidation. Additionally, it has been shown to induce thermogenic genes in murine adipocytes [185]. Follistatin-overexpressing mice showed strong insulin resistance in WAT, increased hepatic glucose production, and glucose intolerance [200], which is in line with the higher levels observed in patients with T2DM. However, higher transient levels of follistatin during and after exercise may actually be beneficial as a whole, favoring continued glucose uptake by SkM and continued WAT lipolysis and FFA uptake by SkM during exercise and post-exercise [4]. Hence, follistatin could persist beyond the acute exercise bout and thus eventually limit TG accretion in WAT by decreasing FFA availability. Nonetheless, this is speculative and should be addressed in future studies.

## 5. The Role of Osteokines in Metabolic Pathways during Exercise

### Osteocalcin

Osteocalcin (OCN) is a bone-derived hormone that is synthesized and secreted by osteoblasts and then activated by osteoclasts during the process of bone resorption [28]. Its importance in metabolism is summarized in Table 3. Ocn**^-/-^** mice presented increased total body fat and hepatic and AT inflammation, along with decreased insulin sensitivity and total energy expenditure [201]. Meta-analyses of clinical studies showed inverse correlations between OCN levels and BMI [202], insulin resistance [203], the inflammatory marker CRP [204], body fat mass [205], and specifically, visceral fat mass, determined with radiologic imaging studies [206,207,208]. Importantly, OCN was independently associated with visceral fat area [206], implying an involvement in the regulation of VAT. OCN promoted the survival and function of pancreatic β-cells and increased insulin secretion [209], while insulin itself induced the release of OCN [210]. OCN may also mediate its beneficial effects by stimulating the synthesis of adiponectin [201,211] and IL-10, by decreasing TNF-α in adipocytes [211] and by upregulating thermogenic genes in AT [212] and mitochondrial biogenesis in SkM [213].

Importantly, OCN is known to increase with acute bouts of exercise, such as 4 × 4-min cycling at 95% maximum heart rate HIIT [28,214]. It directly promotes glucose and FFA uptake by SkM and establishes a positive feedback loop with IL-6, which also results in increased glucose and FFA utilization by SkM and the release of bioactive OCN by bone [71]. Altogether, by stimulating efficient consumption of glucose and FFAs by SkM, as well as by enhancing insulin action directly and via IL-6, OCN decreases available substrates for lipogenesis and at the same time promotes an anti-inflammatory environment that is permissive for thermogenic energy expenditure in AT. These events could indirectly contribute to decreased visceral fat mass, especially considering the strong negative associations between OCN and VAT reported in clinical studies. Further well-designed longitudinal studies that evaluate VAT content in response to changes in OCN levels with long-term exercise interventions are needed.

## 6. The Role of Adipokines in Metabolic Pathways during Exercise

The classic adipokines leptin, adiponectin, and resistin have been extensively reviewed, and their role in the pathogenesis of obesity-related disorders has been concisely outlined. We briefly summarize the physiologic role of each adipokine to highlight their importance in weight and fat mass loss and their maintenance (Table 3). Adipokine levels change in response to chronic exercise rather than to acute bouts of exercise, especially when changes in body composition occur, consistent with the observation that exercise reduces VAT independently of weight loss [6]. Hence, exercise bouts trigger transient changes in other exercise-inducible factors that might mediate metabolic changes and result in decreased WAT mass, shifting the adipokine profile.

### 6.1. Leptin

Leptin is preferentially expressed in subcutaneous WAT and released by adipocytes [69]. As an endocrine signal, leptin suppresses appetite and promotes energy expenditure by activating pro-opiomelanocortin (POMC)-expressing neurons and inhibiting neuropeptide Y (NPY) and agouti-related protein (AgRP) in the hypothalamus. It also enhances SNS outflow to induce thermogenesis [225]. Increased leptin levels have been found in the obese population, inducing a leptin-resistance state, accompanied by increased ROS, TNF-α, IL-6 and chemokine ligands. Additionally, it augments Th1 cytokines IL-2 and IFN-γ while suppressing Th2 cytokine IL-4 [216]. Thus, the hyperleptinemia that accompanies obesity contributes to the establishment of a proinflammatory Th1 cytokine profile and to the persistent M1 Mφ infiltration in obese WAT.

Acute decreases in leptin concentrations in response to acute bouts of aerobic and resistance exercise have been described [217] but, in the presence of an altered hypothalamic set point in obesity, theoretically lead to compensatory overeating and decreased metabolic rate, impairing weight loss [219,236,237]. It appears that in order to restore the physiologic actions of leptin, chronic exercise is needed. Meta-analyses have revealed that chronic aerobic, resistance, and combined exercise resulted in reduced fat mass accompanied by lower leptin concentrations [6,217,219,220]. Improved leptin sensitivity through lower LepR feedback inhibitors, exercise-induced systemic anti-inflammatory environment [9,238], and reduced hypothalamic oxidative stress [220] have been demonstrated. Besides its traditional effect in increasing SNS drive to induce global energy expenditure, restored leptin sensitivity in peripheral organs could also favor maintenance of reduced body fat, as leptin is also known to increase glucose and FFA uptake and oxidation by SkM and to decrease intrahepatic lipid content by promoting FAO [217]. Thus, leptin could also be involved in the redistribution of nutrients away from WAT, which is consistent with its function as an “adipostat” that tightly regulates adipocyte size in physiologic conditions.

### 6.2. Adiponectin

Adiponectin is an adipokine that is almost exclusively expressed by adipocytes in both WAT and BAT [69] and has insulin-sensitizing, anti-inflammatory, and anti-atherogenic properties. When adiponectin binds its receptor ADIPOR1 or 2, downstream activation of AMPK and PPARα ensues, leading to decreased hepatic gluconeogenesis and lipogenesis, increased FAO in the liver and SkM, and increased glucose uptake in SkM and WAT. It directly suppresses the secretion of TNF-α, MCP-1, and IL-6, while increasing IL-10 and the polarization to M2 and reducing β-cell apoptosis [230]. In obesity, adiponectin levels are found to be reduced [216], given that its expression is inhibited by hypoxia, oxidative stress [228], insulin resistance [229], TNF-α, IL-6 [229], “catecholamine resistance” [239], fetuin A [148], and SeP [136].

Regarding exercise, some studies have identified increased adiponectin concentrations during the recovery period following acute aerobic and resistance exercise bouts [217] dependent on exercise intensity [6]. Upregulation by other factors that increase acutely during or shortly after exercise and that have demonstrated in preclinical studies to stimulate the release of adiponectin, such as irisin, IL-15, FGF21, and OCN, must be considered. Regarding chronic exercise, meta-analyses have found that adiponectin levels increase, especially with aerobic long-term interventions in adults across the entire spectrum of BMI, from those with overweight [218] up to those with full-blown T2DM [220]. The increased adiponectin levels induced by exercise are most likely the result of decreased visceral fat mass, improved WAT metabolic status, and a systemic anti-inflammatory environment. In turn, restored levels of adiponectin serve to maintain fat mass in check by contributing to an anti-inflammatory environment permissive for lipolysis, thermogenesis, and its own production, and by increasing oxidative metabolism in SkM and the liver, thereby effectively utilizing excess biofuel.

### 6.3. Resistin

Resistin, although originally identified as an adipocyte-derived adipokine in rodents, is mostly released by monocytes and Mφ in humans [232]. Although its precise mechanisms remain obscure, it is generally agreed upon that its expression is induced by proinflammatory stimuli and that it in turn perpetuates the proinflammatory response and insulin resistance by interacting with TLR4 [69].

Controversy exists as to the role of exercise in its regulation in patients with obesity. Some have either shown a reduction in resistin levels [233] or no change [227] following aerobic or resistance exercise interventions. Some authors have recently proposed that the exercise-induced reduction of resistin levels in patients with T2DM might be due to the anti-inflammatory milieu that exercise elicits, rather than the change in BMI [233]. However, the systemic anti-inflammatory environment may be attributed to decreased adiposity [9], which cannot always be appreciated with a change in BMI without exploring other adiposity parameters.

## 7. An Integrative Overview of Exercise-Induced Factors Influencing Appetite Regulation, Mechanisms for Energy Expenditure, Fat Mass Loss, and Healthy Weight

Physiologic responses persist beyond an acute exercise bout and during the recovery period. For instance, after the immediate increase, lipolysis in WAT stabilizes but remains higher than the baseline rate for up to 24 h post-exercise [18,240], while the increased BMR may persist for up to 48 h [33]. The effects during the post-exercise period are likely to be mediated by changes in the levels and/or activity of many exercise-inducible factors.

### 7.1. Influence of Exercise on the Central Control of Energy Expenditure and Appetite

**Figure 1:**
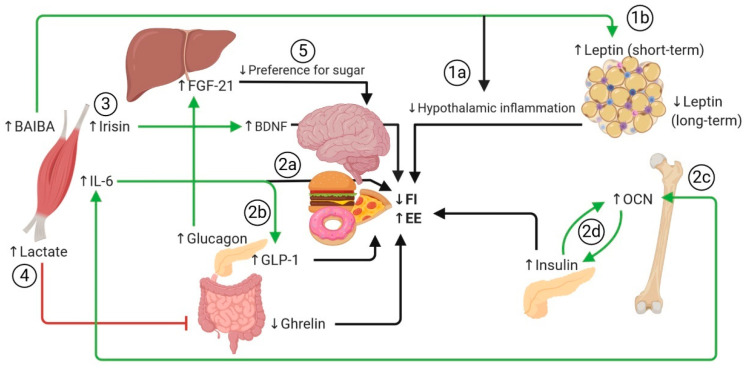
Effects of exercise-inducible factors on appetite control. Details in text. Abbreviations: BAIBA: β-Aminoisobutyric acid; EE: Energy expenditure; FGF21: Fibroblast-growth factor 21; FI: Food intake; GLP-1: Glucagon-like peptide 1; IL-6: Interleukin-6; OCN: Osteocalcin. **Green arrows** represent a stimulatory effect over another mediator; **red inhibitor lines** represent an inhibitory effect over another mediator; **black arrows or inhibitor lines** indicate the final physiologic effect of any given mediator, either stimulatory or inhibitory, respectively. Custom image created with Biorender.

The physiological effects induced by exercise may counter central neuroendocrine adaptive responses that favor weight regain [241,242]. Through diverse mechanisms, exercise-inducible factors may directly or indirectly modulate appetite regulation and energy expenditure in the CNS (Figure 1; *to facilitate sequence flow, the steps appear numbered as they are introduced in the main text in italics*). Importantly, appetite homeostasis is known to be altered in obesity due at least in part to hypothalamic inflammation and reactive gliosis that results in functional impairment of anorexigenic neurons and central leptin and insulin resistance [243,244]. Animal studies have shown that endurance exercise increases the phosphorylation of hypothalamic JAK2, STAT3, Akt, and Erk involved in the leptin signaling pathway [245], which could be directly mediated by organokines [7]. These mechanisms contribute to loss of fat mass and, in time, to its maintenance. First, exercise-induced BAIBA has been recently shown to directly inhibit hypothalamic inflammatory reactions in mice fed a high-fat diet [114], which could indirectly increase central sensitivity to anorexigenic hormones [243] *(1a)* and could also be a complementary mechanism to its own ability to stimulate leptin production by adipocytes [109] *(1b).* Second, IL-6 released by SkM, directly *(2a)* or indirectly via GLP-1 *(2b)*, may also have central anorexigenic properties by modulating the activity of hypothalamic neurons involved in appetite regulation [7,122]. Moreover, IL-6 increases production of OCN by osteoblasts [71] *(2c),* which itself stimulates transient pancreatic secretion of the anorexic hormone insulin and triggers a positive feed-forward mechanism that results in increased OCN and thus more insulin [28,209,210] *(2d).* Third, exercise-induced irisin may promote brain-derived neurotrophic factor (BDNF) synthesis in the CNS [7,67,68]; BDNF is a peptide known to possess central anorexigenic effects and to promote increased energy expenditure by increasing sympathetic outflow [246] *(3).* Fourth, production of lactate by SkM during high-intensity exercise may inhibit production of ghrelin, further contributing to decreased hunger [247] *(4).* Finally, increased levels of the hepatokine FGF21, although not influencing appetite per se, have been linked to decreased intake of obesogenic sugary foods [168] *(5).* Overall, reduced hypothalamic inflammation, release of osteokines and myokines, and increased sensitivity to anorexigenic hormones induced by exercise lead to reduced caloric intake and loss of fat mass. In the long term, reduced circulating leptin associated with decreased fat mass and an anti-inflammatory environment may help restore hypothalamic leptin (and insulin) sensitivity, thus restoring the balance between energy expenditure and food intake.

### 7.2. Mechanisms Through which Exercise-Induced Factors Exert Anti-Inflammatory Effects and the Influence of These Effects on Insulin Sensitivity and Loss of Fat Mass

**Figure 2:**
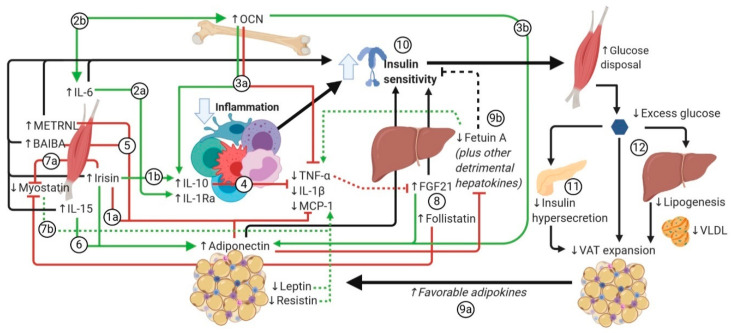
Effects of exercise-inducible factors on the systemic inflammatory milieu and insulin sensitivity. Details in text. Abbreviations: BAIBA: β-Aminoisobutyric acid; FGF21: Fibroblast growth factor 21; IL-1Ra: Interleukin-1 receptor antagonist; IL-1β: Interleukin-1β; IL-6: Interleukin-6; IL-10: Interleukin-10; IL-15: Interleukin-15 MCP-1: Monocyte chemoattractant protein-1; METRNL: Meteorin-like; OCN: Osteocalcin; TNF-α: Tumor necrosis factor-alpha. **Green arrows** represent a stimulatory effect over another mediator; **red inhibitor lines** represent an inhibitory effect over another mediator; **black arrows or inhibitor lines** indicate the final physiologic effect of any given mediator, either stimulatory or inhibitory, respectively; **dotted** lines indicate absence of an expected effect, either stimulatory or inhibitory. Custom image created with Biorender.

In obesity, especially in visceral adipose tissue (VAT), the initial adipocyte hypertrophy triggers adipocyte hypoxia and mechanical stress, which further results in adipocyte necrosis, all of which elicit a proinflammatory response mediated by damage-associated molecular patterns (DAMPs). The release of MCP-1 by stressed adipocytes has been shown to trigger WAT infiltration of monocytes that polarize to the classically activated M1 phenotype. M1 macrophages secrete TNF-α, IL-1β, IL-6, and chemokines that promote the influx of activated CD4+ Th1 cells [14,20,239,248]. This response is accompanied by local insulin resistance due to activation of kinases that phosphorylate serine residues of IRS1 and by excessive generation of ROS. A chronic systemic inflammatory state ensues, which in the presence of insulin resistance, promotes ectopic accumulation of lipids in the liver and SkM that exacerbates the inflammatory response, while at the same time promoting a state of “catecholamine resistance” in WAT. Proinflammatory cytokines such as TNF-α and IL-1β exacerbate insulin resistance [20], while anti-inflammatory cytokines such as IL-4 and IL-10 promote insulin sensitivity [248]. Exercise likely plays a role in the maintenance of an anti-inflammatory microenvironment and may help reverse WAT abnormalities observed in obesity. Although acute exercise was found to trigger a transient increase in oxidative stress and proinflammatory cytokines, chronic exercise was shown to induce a systemic anti-inflammatory response not only in the recovery period but also in the long term, an effect hypothesized to partially mediate its health benefits [9,238]. In healthy individuals subjected to aerobic exercise interventions, systematic reviews and meta-analyses have found decreased circulating levels of TNF-α, IL-6, and CRP [249], as well as increased levels of the anti-inflammatory cytokine IL-10 [250]. A meta-analysis focusing specifically on individuals with T2DM has also described decreased levels of IL-6 and CRP with aerobic exercise interventions [227], which emphasizes that the anti-inflammatory properties of exercise are strong regardless of preexisting metabolic status. The systemic anti-inflammatory milieu in individuals who exercise regularly is also evidenced by decreased circulating inflammatory monocytes, which also show decreased expression of TLRs on their surface, increased IL-10-producing circulating regulatory T-cells (T^regs^), and a shift in WAT immune cell infiltrates favoring a higher M2:M1 Mφ ratio [9]. Physical activity likely plays a determinant role in the release of many factors that synergize to evoke a systemic anti-inflammatory response, both in the short and long term (Figure 2). A summary of the immune cytokines affected by exercise is given in Table 4. By generating an anti-inflammatory microenvironment in VAT, catecholamine- and exercise-factor-induced sensitivity to lipolysis could be restored. Thus, released FFAs from this depot could be effectively redistributed to the SkM (which has undergone structural and biochemical adaptation for their utilization), which at the same time decreases availability of FFAs that can eventually be re-esterified and reincorporated into VAT [251]. Furthermore, considering that BAT function is dependent on an anti-inflammatory environment [12], this could also restore its thermogenic capacity, leading to enhanced energy expenditure and, consequently, loss of fat mass. These end-effects could be of particular relevance in humans given that VAT adipocytes have higher expression of β3 adrenergic receptors than those in scAT [252].

As also shown in Figure 2 (*to facilitate sequence flow, the steps appear numbered as they are introduced in the main text in italics*), in the short term, irisin might directly favor a shift from M1 Mφ to a M2 phenotype [49,59], as well as decrease the expression of TLR4, TNF-α, IL-1β, MCP-1, and IL-6 by adipocytes and Mφ [51,53,59] *(1a)*, while increasing IL-10 [59] *(1b)* and antioxidant cellular mechanisms [60]. On the other hand, IL-6 has been shown to inhibit the transcription of IL-1 and TNF-α [74] while stimulating the release of IL-1Ra, IL-10, and cortisol in human mononuclear cells [72] *(2a).* Additionally, it has been found to upregulate the secretion of OCN by bone [71] *(2b),* which reduces the expression of TNF-α and increases that of IL-10 *(3a)* and adiponectin in adipocytes [211] *(3b).* IL-10, in turn, was found to inhibit the expression of proinflammatory cytokines [9], while IL-1Ra was shown to antagonize the actions of IL-1β [8] *(4).* Similarly, the myokines METRNL [100] and BAIBA [110] have proven to suppress TNF-α and other proinflammatory cytokines both in vitro and in vivo *(5).* In addition, METRNL was able to indirectly stimulate M2 Mφ polarization by inducing eosinophils to secrete IL-4 and IL-13 [98]. Meanwhile, IL-15 was noted to antagonize the detrimental actions of TNF-α, considering its antioxidant properties [26,87] and its ability to stimulate adiponectin release [86] *(6).* Likewise, exercise-induced reduction of myostatin by irisin [44] *(7a)* was described to reduce availability of myostatin to engage in proinflammatory responses [116] *(7b).* Moreover, the hepatokines FGF21 and follistatin, which are induced acutely by exercise, were found to contribute as well. FGF21 was determined to increase glucocorticoid action [164] and the secretion of adiponectin [165], while follistatin was found to bind and inactivate myostatin [184] *(8)*, further inhibiting its proinflammatory effects. In the long term, reduced WAT depots by exercise favor a shift in the adipokine profile [218,219,220] *(9a)*, and possibly the hepatokine profile *(9b)*, due to the reduction of hepatic steatosis that occurs concomitantly with weight loss [23]. Altogether, increased adiponectin and decreased leptin from WAT, as well as decreased proinflammatory hepatokines fetuin A and SeP, among others, may help maintain the anti-inflammatory state and thus insulin sensitivity *(10*).

Enhanced insulin sensitivity could help inhibit fat mass expansion by two mechanisms. The first mechanism is by decreasing the compensatory hyperinsulinemic response that occurs with insulin resistance *(11).* In this regard, hyperinsulinemia has been proposed to be an early crucial driver in the progression of adiposity [265,266,267,268]. Insulin has been shown to activate genetic programs involved in adipogenesis in genetically modified mice [266]. In another study, mice with partial insulin deficiency were found not to develop hyperinsulinemia following exposure to a high-fat diet (HFD), while displaying resistance to weight gain and adiposity, compared with wild-type littermates [269]. Moreover, WAT-specific insulin receptor deficiency was shown to protect mice from obesity, independently of food intake [270,271]. In humans, the relevance of this concept is supported by the observation that hyperinsulinemia following weight loss is predictive of weight regain [272]. Regarding exercise interventions, clinical studies have found decreased fasting levels of insulin not only in healthy individuals who engage in supervised exercise interventions [35], but also in patients with established T2DM at the other end of the spectrum [273]. As the second mechanism, enhanced insulin sensitivity can inhibit the further expansion of VAT by redirecting excess glucose into the increased exercise-adapted SkM mass (where it can be consumed as metabolic fuel or stored as glycogen in the resting state) and away from hepatic lipogenesis (and thus, eventual TG accretion in WAT through VLDLs) *(12).* This is particularly relevant when considering that insulin resistance may occur to a greater degree in the liver than in WAT in metabolic disease [268], thus perpetuating hepatic lipogenesis, hypertriglyceridemia, and excessive fat deposition in WAT, exacerbated by exaggerated LPL activity induced by hyperinsulinemia. Importantly, some exercise-induced factors have also been shown to have direct insulin-sensitizing effects in target tissues, independently of their effect on inflammation, such as irisin [56], IL-6 [122], IL-15 [84], METRNL [274], BAIBA [112], and FGF21 [154] *(10).* Concurrently, other exercise-induced factors, such as irisin [66], follistatin [183], and OCN [209], directly protect β-cells from damage, further potentiating their beneficial metabolic properties. Altogether, decreased insulin resistance and concomitant decreased β-cell stress elicited by exercise-induced factors could result in mitigation of hyperinsulinemia and decreased availability of lipogenic substrate, hence reducing lipogenesis in susceptible WAT depots and thus reducing WAT expansion (Figure 2). In summary, it could be agreed that repeated exercise bouts result in an accumulative effect on inflammation and metabolism, while chronic exercise maintains the resulting basal anti-inflammatory, insulin-sensitive state that favors physiologic fat mass maintenance.

### 7.3. Mechanisms of Exercise-Induced Factors on WAT Browning in Health and in Obesity

Exercise itself increases total-body thermogenic activity and energy expenditure, highlighting the counterintuitive impression that exercise could mediate WAT browning in a situation where preservation of energy would be an expected compensatory response. This is known as the “exercise-induced browning paradox”. This apparently paradoxical response may be a defense mechanism against ectopic accumulation of lipids. During exercise, lipolysis leads to increased circulating FFAs, which, if not expended as metabolic fuel in the liver or SkM, would be stored in tissues, leading to lipotoxicity with its associated detrimental consequences, such as oxidative stress, inflammation, and insulin resistance. Thus, by inducing WAT browning, exercise increases the total-body capacity to oxidize FFAs, thereby counteracting ectopic lipid accumulation [79]. A second hypothesis posits that exercise reduces body insulation by decreasing adipocyte size and lipid content in scAT and thus activates thermogenic processes to increase body temperature as a compensatory mechanism [16,79]. Increased WAT browning would be expected to increase BMR, a plausible mechanism through which exercise induces fat loss and weight loss.

**Figure 3:**
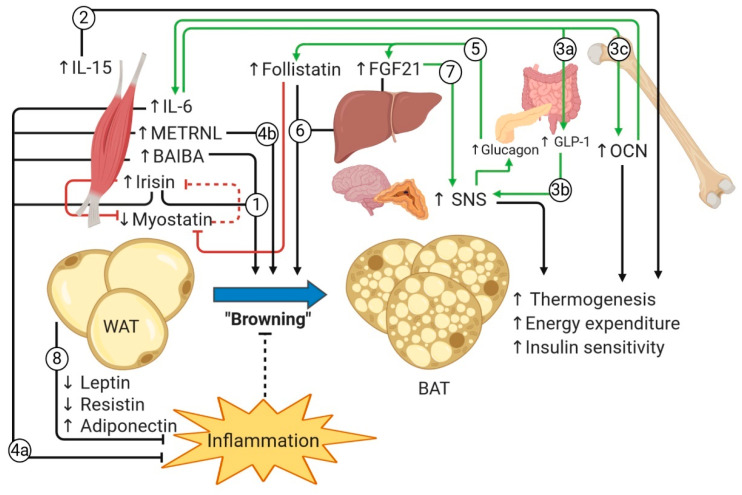
Effects of exercise-inducible factors on WAT “browning”. Details in text. Abbreviations: BAIBA: β-Aminoisobutyric acid; BAT: Brown (and beige) adipose tissue; FGF21: Fibroblast growth factor 21; GLP-1: Glucagon-like peptide 1; IL-6: Interleukin-6; IL-15: Interleukin-15; METRNL: Meteorin-like; OCN: Osteocalcin; SNS: Sympathetic nervous system; WAT: White adipose tissue. **Green arrows** represent a stimulatory effect over another mediator; **red inhibitor lines** represent an inhibitory effect over another mediator; **black arrows or inhibitor lines** indicate the final physiologic effect of any given mediator, either stimulatory or inhibitory, respectively; **dotted** lines indicate absence of an expected effect, either stimulatory or inhibitory. Custom image created with Biorender.

Preclinical studies, mostly in rodents, have shown that WAT browning can be generated by various exercise-inducible factors, as shown in Figure 3 (*to facilitate sequence flow, the steps appear numbered as they are introduced in the main text in italics*). The myokines irisin [57] and BAIBA [104] have been described to directly induce WAT “browning” both in vitro and in vivo, by upregulating genes involved in mitochondrial biogenesis, FAO, oxidative phosphorylation, and thermogenesis *(1).* In addition, IL-15 was found to enhance the activity of BAT in rodents [275] *(2).* SkM-derived IL-6 was described to stimulate the release of GLP-1 [73] *(3a)*, enhancing SNS output [19] *(3b)*, and to promote the release of OCN [71] *(3c*), which in turn upregulates PGC-1α and UCP-1 in adipocytes [212]. Importantly, as stated above, some of these myokines also possess anti-inflammatory properties *(4a)*, which is of relevance given that an anti-inflammatory environment is optimal for differentiation and activity of BAT [12], as exemplified by METRNL, which has been found to indirectly promote WAT browning by favoring M2 polarization of Mφ [98] *(4b).* Additionally, the exercise-induced hepatokines FGF21 [43] and follistatin [185], both upregulated by glucagon (*5*), have also been described to induce WAT browning directly (*6*). FGF21 further augments BAT function indirectly, by increasing SNS output [163,165] *(7)*, and possibly mediates an hepatokine–myokine crosstalk via activation of PGC-1α [43], which is known to upregulate the WAT browning myokines FNDC5-irisin [57], METRNL [274], and BAIBA [104]. In the long term, exercise-induced loss of fat mass may favor a permissive environment for WAT browning and BAT function by decreasing the expression of proinflammatory adipokines and increasing that of adiponectin [6] *(8*), thus maintaining a beneficial feed-forward cycle that promotes redistribution and consumption of excess hydrocarbon biofuel. This is consistent with the fact that BAT can indeed participate in the uptake and metabolism of circulating glucose and FFAs [17]. Hence, excess diet-derived glucose or FFAs could also be transported for their disposal to these specialized adipose depots along with exercise-adapted SkM, thus decreasing available substrates for lipogenesis in the liver and WAT expansion. In fact, the greatest benefits of BAT have been suggested to result from the redistribution of nutrient disposal rather than from thermogenesis per se [34]. BAT has the particular property that, although FFAs from stored TGs are oxidized, the presence of UCP1 uncouples the electron transport chain from ATP synthesis, thus decreasing available ATP for anabolic processes (e.g., re-esterification of FFAs into TGs, which may occur in WAT) and instead dissipating chemical energy as heat. Thus, BAT catabolizes metabolic substrates in “futile cycles”, resulting in increased energy expenditure and net loss of fat mass.

Although well-validated in rodents, exercise-induced WAT browning remains controversial in humans [16]. Most studies have not found evidence of WAT browning when applying various types and durations of exercise interventions [79]. Nonetheless, increased expression of BAT genes in scAT of adults (across a wide BMI range) subjected to an aerobic exercise program has recently been demonstrated [276]. Two hypotheses have been formulated based on differences in WAT physiology between mice and humans. First, exercise-induced browning might occur in different WAT depots in humans than those in mice. Most studies in humans have evaluated the browning response in scAT biopsies, which at a first glance seems appropriate, given that it is the main AT depot subject to browning in mice [79]. However, a recent study has determined that humans express more BAT-related genes in VAT, while mice express these genes in scAT to a greater degree [277]. Second, the relatively low levels of β-adrenergic receptor expression in human scAT compared with those in VAT [252] could account for the lack of exercise-induced browning. Thus, the lack of browning in humans might be due to research not being focused in the appropriate location. If indeed BAT is induced to a greater degree in VAT in humans, then this could represent a potential mechanism for the decreased VAT mass observed with exercise.

Another interesting possibility is that the lack of observable browning in obese populations might represent a pathophysiological phenomenon, rather than a lack of such an effect [13], given that browning in VAT has not been studied in this population and that any detectable effect is likely dampened by the proinflammatory milieu in obesity. Supporting this hypothesis, “catecholamine resistance” is known to occur in individuals with long-standing obesity. Particularly, lipolysis and activation of transcription factors that result in WAT browning require signaling through the β3-adrenergic receptor. Its downstream signaling pathway may be attenuated in obesity by chronic exposure to TNF-α through NF-κB-mediated upregulation of IKKε and TBK1, which inhibit cAMP signaling via phosphodiesterase 3B [239]. Furthermore, M2 anti-inflammatory Mφ, mostly absent in obese WAT, appear to be crucial in the process of WAT browning [12], via as of yet unclear mechanisms. In addition, M1 proinflammatory Mφ, abundant in obese WAT, seem to inhibit beige adipogenesis. In line with this, an inflammatory WAT microenvironment was shown to impair beige adipogenesis through contact-dependent interactions between M1 Mφ and adipocytes in a murine model. TNF-α was shown to upregulate VCAM-1 in adipocytes, which induced direct adhesion to M1 Mφ via α_4_ integrin. Additionally, TNF-α decreased UCP1 expression in adipocytes by an Erk-dependent mechanism when cultured in the presence of browning agents NE and T3. A similar mechanism was found in human adipocytes as well [278]. This highlights the possibility that chronic exposure to an overwhelming inflammatory milieu could impair browning in humans. If indeed exercise-induced WAT browning were to occur in the visceral compartment to a greater degree in humans, this would theoretically dampen the loss of visceral fat mass. If this were the case, then it could help explain why obese subjects have suboptimal results in achieving weight loss when the only adopted lifestyle modification is exercise [279].

Alternatively, impaired endocrine regulation of exercise-induced factors involved in WAT browning could represent yet another possibility in the context of obesity. Clinical studies in patients with insulin resistant conditions have described dampened upregulation of exercise-induced factors, such as FGF21 and follistatin, most likely due to an altered glucagon-to-insulin ratio [156,157,280], and possibly irisin, which is known to be downregulated by glucotoxic, lipotoxic [47,48], and inflammatory conditions [45]. The opposite may occur for fetuin A, whose lack of exercise-induced decrease might be explained by its upregulation in the context of insulin resistance [144,145]. Concomitantly, resistance to release of exercise-inducible factors might also be involved, potentially elucidating, at least in part, why FGF21, follistatin, irisin, and METRNL are frequently increased in patients with obesity and T2DM, even in basal conditions.

### 7.4. Effects of Exercise-Induced Factors on Lipolysis, Fatty Acid Oxidation (FAO), Reduced Lipogenesis, and the Redistribution of Fatty-Acid-Derived Energy Fuel

**Figure 4:**
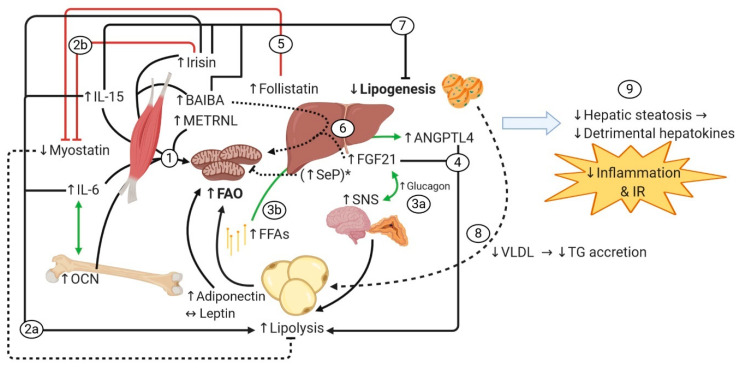
Effects of exercise-inducible factors on hepatic and SkM FAO, WAT lipolysis, and hepatic lipogenesis. Details in text. *Although SeP is not regulated by exercise, its high concentrations in obesity might impair SkM adaptation to oxidative metabolism. Abbreviations: ANPTL4: Angiopoietin-like 4; BAIBA: β-Aminoisobutyric acid; FAO: Fatty acid oxidation; FFAs: Free fatty acids; FGF21: Fibroblast growth factor 21; IL-6: Interleukin-6; IL-15: Interleukin-15; IR: Insulin resistance; METRNL: Meteorin-like; OCN: Osteocalcin; SeP: Selenoprotein P; TG: Triglycerides; VLDL: Very-low-density lipoprotein. **Green arrows** represent a stimulatory effect over another mediator; **red inhibitor lines** represent an inhibitory effect over another mediator; **black arrows or inhibitor lines** indicate the final physiologic effect of any given mediator, either stimulatory or inhibitory, respectively; **dotted** lines indicate absence of an expected effect, either stimulatory or inhibitory. Custom image created with Biorender.

The total-body FAO rate is determined not only by AT but also by metabolic activity in the SkM and in the liver. Glucose and FFAs have been shown as metabolic substrates for the SkM when an individual is engaged in aerobic exercise [30], but FFAs become the main substrate in the post-exercise recovery period [32], depending on both the SkM enzymatic oxidative capacity and substrate availability supplied largely by WAT and the liver. Following bouts of aerobic exercise, circulating FFAs and the SkM FAO remain significantly elevated depending on exercise intensity and duration [32]. Aerobic exercise influences these processes, at least in part, through the action of exercise-inducible factors, as summarized next and illustrated in Figure 4 (*to facilitate sequence flow, the steps appear numbered as they are introduced in the main text in italics*).

Figure 4 shows the effects of exercise-inducible factors on hepatic and SkM FAO, WAT lipolysis, and hepatic lipogenesis. Exercise-inducible myokines, such as irisin [62,63], IL-6 [76,77], IL-15 [91,126], BAIBA [111], and METRNL [100], as well as the osteokine OCN [71] have been shown to directly stimulate oxidative metabolism in autocrine and endocrine ways, respectively *(1).* These mechanisms include the upregulation of transporter molecules involved in glucose and FFA uptake and of the enzymes involved in FAO, along with the stimulation of mitochondrial biogenesis, which enhances the oxidative capacity of the SkM. Furthermore, some myokines could contribute to the WAT lipolysis in an endocrine fashion. For instance, irisin [58], IL-15 [89], and IL-6 [75] have shown to directly induce lipolysis in adipocytes *(2a)*, while irisin exerts a dual effect by also inhibiting myostatin [44] (*2b*), reducing myostatin-mediated inhibition of WAT lipolysis [132]. The liver also contributes by releasing hepatokines, namely FGF21, follistatin, and ANGPTL4, during exercise, with glucagon as the main prompter *(3a)*, as well as in the recovery period, in which circulating FFAs are the chief driver via PPAR-α [3,4] *(3b).* While both FGF21 [166] and ANGPTL4 [176,177] induce WAT lipolysis *(4)*, the latter may selectively inhibit WAT LPL to favor lipoprotein-derived FFAs by the exercising SkM [197]. Follistatin, on the other hand, by inhibiting myostatin [184] *(5)*, might also contribute to continued lipolysis in WAT. Importantly, these exercise-inducible factors remain in circulation for hours after the exercise bouts, and thus their effects in target tissues can be expected to persist beyond the acute exercise bout. In brief, myokines and hepatokines, by stimulating oxidative metabolism concomitantly with lipolysis in the post-exercise period, increase the proportion of FFAs that are oxidized instead of being re-esterified into TGs that are eventually returned to WAT, thus limiting WAT expansion, possibly in visceral depots.

Exercise also exerts beneficial effects by decreasing fatty accumulation in the liver. Hepatic steatosis, present in up to 70% of individuals with overweight and in >90% of morbidly obese ones [23], has been shown to perpetuate obesity and the systemic proinflammatory and insulin-resistant state. Impaired hepatic FAO (most likely due to mitochondrial dysfunction in hepatocytes), increased circulating FFAs in the setting of basal VAT lipolysis (secondary to full-blown insulin resistance), and exaggerated lipogenesis, are considered pathogenic hallmarks of hepatic steatosis [23]. Exercise might importantly target these processes through exercise-inducible factors, which might be responsible for the beneficial effects, as supported by a recent meta-analysis that described reduced intrahepatic TG content in humans subjected to aerobic exercise interventions [24]. For instance, BAIBA [104] and FGF21 [160] have been shown to increase hepatic β-oxidation *(6*), while others such as irisin [65], IL-15 [90], and BAIBA [113] have been found to inhibit hepatic lipogenesis *(7).* OCN [28,215] and irisin [61,65], on the other hand, may also exert direct anti-oxidant and anti-inflammatory effects in the liver. These effects might be clinically relevant, as an inverse association between irisin concentrations and hepatic TG content in adults has been recently reported [281]. The added-up effects of the exercise-inducible factors could prevent hepatic lipid accumulation by increasing their oxidation and reducing lipogenesis, which would in turn decrease the accretion of TGs in WAT via LPL, thereby inhibiting WAT expansion *(8).* A synergistic effect in the SkM after exercise, mediated by ANGPTL4, might occur. In addition, this might indirectly result in a more favorable hepatokine profile *(9).* This is supported by clinical studies that have found that exercise interventions decrease the levels of detrimental hepatokines and increase the metabolically favorable ones, counteracting systemic inflammation and insulin resistance, as well as eliciting an increase in global energy expenditure.

In summary, through the summed metabolic effects of a favorable hepatokine profile, redistribution of hydrocarbon fuel sources is favored towards catabolism in SkM and BAT and away from anabolic pathways in the liver and eventually in WAT, thus potentially impairing WAT expansion in visceral depots. Increased circulating FFAs due to myokine- and hepatokine-induced lipolysis synergize with the lipolytic effects of catecholamines in WAT and, in conjunction with myokine- and hepatokine-induced FFA uptake and oxidation by SkM and the liver, may contribute to limit the availability of circulating FFAs for lipogenesis in WAT, thereby explaining the decreased visceral adiposity observed in preclinical models. In the long term, loss of fat mass also triggers favorable changes in the adipokine profile, augmenting adiponectin levels and further favoring FAO in SkM and the liver, as previously described.

### 7.5. Considerations for Exercise-Induced Fat Mass Loss and Weight Loss in the Obese State

Overall, most clinical evidence to date supports the concept that exercise is more effective in preventing overweight and obesity than in their reversal [279]. Nevertheless, it is important to highlight that exercise has been shown to reduce VAT depots independently of overall weight changes, and strong evidence suggests that it is more effective than hypocaloric dietetic interventions. In fact, weight loss, as a single measurement of the efficacy of an exercise intervention, is an inappropriate and misleading indicator [282], as exercise not only induces VAT loss but may also promote gain of fat-free mass [283], balancing the total weight. Thus, the importance of exercise should not be overlooked even when it does not result in net weight loss [241]. However, some physiological differences must be considered when analyzing the fat-reducing role of exercise in the context of obesity.

Obesity unleashes a self-perpetuating cascade of detrimental mechanisms that result in the maintenance of obesity itself and in the appearance of metabolic complications. Concretely, detrimental neuroendocrine positive feedback loops that are initiated and maintained in the obese state may be part of the explanation for why, once established, excess weight may prove difficult to lose if the only adopted lifestyle modification is exercise at the currently recommended levels of 150 min/week. In fact, evidence has shown that a higher exercise duration is needed to reverse obesity than to maintain a healthy weight [279]. Previously obese or overweight individuals were shown to require higher duration of exercise to maintain a healthy weight (200–300 min/week of moderate-intensity aerobic exercise) compared with those who were not previously overweight (150–250 min/week) [284]. This relative difficulty in attaining weight loss with preexisting obesity may be related to the combination of several mechanisms, such as an overwhelming proinflammatory milieu resulting in a persistently altered hypothalamic sensitivity to appetite hormones, imbalanced energy expenditure, increased insulin resistance, hyperinsulinemia leading to enhanced adipogenesis, and impaired WAT “browning”. In addition, resistance or impaired synthesis of exercise-inducible factors in the obese population could be contributors to weight-loss-resistance, requiring higher intensity, frequency, and/or duration of exercise than what is commonly prescribed in order to be released to an extent significant enough to reduce fat mass. Indeed, an intensity-dependent increase in the levels of FGF21 and follistatin in healthy untrained adults subjected to a 45-min treadmill challenge has been recently described [285]. Furthermore, clinical trials with a synthetic FGF21 analog effectively reduced weight and increased insulin sensitivity in obese individuals [286]. Thus, increased concentrations of these exercise-inducible factors could hypothetically overcome the already established resistance to the favorable effects of these factors in obesity. Resistance to the effects of exercise-inducible factors on lipid mobilization and redistribution of hydrocarbon fuel away from WAT and its effect on visceral fat mass is an appealing area of study and warrants further investigation.

### 7.6. Exercise Promotes Decreased Fat Mass, Healthy Weight, and Balanced Energy Metabolism by Exercise-Induced Factors: Putting it All Together

**Figure 5:**
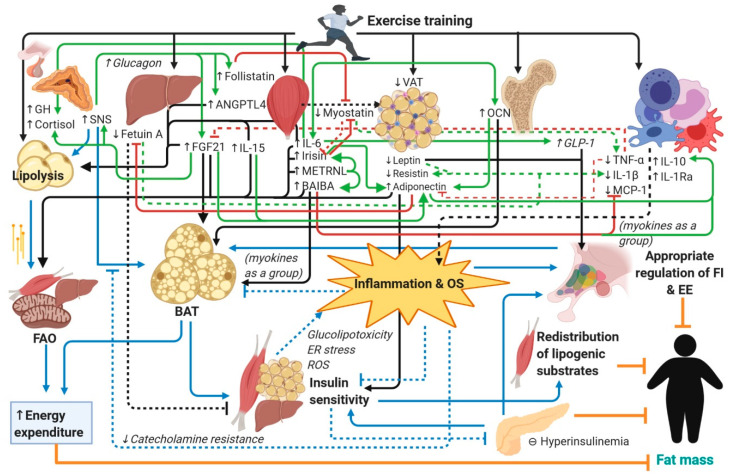
An integrated view of the role of exercise training on pathways associated with energy expenditure, fat mass loss, redistribution of energy substrates, adipose tissue reserves, and immunometabolic health: integrated neuroendocrine pathways associated with exercise training that affect global AT reserves and function. Interdependence between the processes of central control of energy balance, WAT browning, inflammation, and insulin sensitivity is highlighted. AT: adipose tissue, ANGPTL4: Angiopoietin-like 4; BAIBA: β-Aminoisobutyric acid; BAT: Brown (and beige) adipose tissue; EE: Energy expenditure; ER: Endoplasmic reticulum; FAO: Fatty acid oxidation; FI: Food intake; FGF21: Fibroblast growth factor 21; GH: Growth hormone; GLP-1: Glucagon-like peptide 1; IL-1Ra: Interleukin-1 receptor antagonist; IL-1β: Interleukin-1β; IL-6: Interleukin-6; IL-10: Interleukin-10 IL-15: Interleukin-15; MCP-1: Monocyte chemoattractant protein-1; METRNL: Meteorin-like; OCN: Osteocalcin; OS: Oxidative stress; ROS: Reactive oxygen species; SNS: Sympathetic nervous system; TNF-α: Tumor necrosis factor alpha; VAT: Visceral adipose tissue. **Green arrows** represent a stimulatory effect over another mediator; **red inhibitor lines** represent an inhibitory effect over another mediator; **black arrows or inhibitor lines** indicate the final physiologic effect of any given mediator, either stimulatory or inhibitory, respectively; **blue arrow or inhibitor lines** indicate the consequence of a process as a whole, either stimulatory or inhibitory, respectively; **orange inhibitor lines** indicate the final processes in the control of fat mass; **dotted lines** indicate absence of an expected effect, either stimulatory or inhibitory. Custom image created with Biorender.

Figure 5 shows an integrated view of the role of exercise training on pathways associated with energy expenditure, fat mass loss, redistribution of energy substrates, adipose tissue reserves, and immunometabolic health. Altogether, accumulative bouts of aerobic exercise promote the release of exercise-inducible factors that interact with one another (Table 5), convey direct insulin-sensitizing and anti-inflammatory properties, and favor metabolic adaptations that increase oxidative metabolism, jointly resulting in decreased body fat mass. The end effects of the main organokines are summarized and compared in Table 6. Decreased fat mass is accompanied by favorable changes in the adipocytokine, hepatokine, and myokine profiles. These, in turn, further maintain a systemic anti-inflammatory environment, subsequently promoting insulin sensitivity and a rebalanced total energy expenditure. This is accomplished by reversal of BAT dysfunction and a finer regulation of the hypothalamic circuitry controlling energy expenditure and food intake. Additionally, the decreased fat mass and insulin sensitivity associated with exercise, along with the direct actions on the liver of the exercise-inducible factors, lead to resolution of fatty liver, which in turn could help restore a healthy hepatokine profile that further enhances immunometabolic health. Although these proposed mechanisms are indeed interesting and important, more research is needed to corroborate these hypotheses, which are summarized in Figure 5.

## 8. Conclusions and Future Directions

Exercise unleashes a complex network of endocrine interactions in which circulating factors, released in response to exercise, interplay through inter-organ crosstalk and physiologic changes. Exercise influences a favorable organokine profile that, per se, mediates many of its beneficial health effects. In summary, acute exercise bouts appear to modulate the release of myokines, hepatokines, osteokines, and immune cytokines, while chronic exercise training correlates with changes in basal circulating adipokines and immune cytokines, possibly in association with weight loss itself. In conjunction, organokines act in an orchestrated manner to modulate systemic metabolic processes. Their concentrations and effects change in response to the intensity, duration, and frequency of exercise to directly or indirectly control WAT reserves. Beneficial organokines induced by exercise include irisin, IL-6, IL-15, METRNL, BAIBA, FGF21, ANGPTL4, follistatin, osteocalcin, and adiponectin, which jointly mediate the mechanisms and effects. These include the following: (1) SkM adaptation to exercise, including hypertrophy and induction of catabolic enzymes, thus promoting efficient glucose and FFA utilization that precludes these energetic fuels from entering anabolic pathways in the liver and WAT; (2) browning of WAT, which, in addition to SkM, dissipates the energy coming from the excess of nutrients in the form of heat; (3) induction of an anti-inflammatory and insulin-sensitive state that is permissive for physiologic lipolysis induced by catecholamines or other exercise-inducible factors in response to exercise and BAT activity; (4) redistribution of energy fuel away from WAT and available for SkM, as well glucose disposal in SkM, thus decreasing available substrate for lipogenesis; and (5) a restored control of energy balance by the CNS. Furthermore, in the long term, decreased levels of detrimental hepatokines such as selenoprotein P and fetuin A, as well as normalized expression of leptin and decreased levels of resistin, further aid in maintaining healthy weight and fat mass by decreasing systemic inflammation and insulin resistance, thus being permissive to all of the above mechanisms. Considering the potent synergistic and pleiotropic effects of exercise-inducible factors summarized in Figure 5, it is plausible that, in addition to protecting against the development of obesity, exercise, when performed at a sufficient frequency, intensity, and duration, is a powerful tool for dissipating excess visceral fat mass that is causally involved in the pathogenesis of chronic cardiometabolic diseases.

Important areas to address in future research include the translational potential of the preclinical evidence, the identification of relevant effects of exercise-inducible factors from a clinical perspective, and, finally, the inter-individual variability of the physiologic response to exercise interventions, especially in obese populations. Specifically, considering that groups of exercise-inducible factors have been mostly studied independently, future interventional clinical studies with large sample sizes should address a comprehensive panel to evaluate the status of all groups of organokines, ideally at the tissue level (when feasible) and in circulation. This type of research ought to be carried out before and after predetermined time intervals following acute exercise bouts and chronic training regimens of different modalities, durations, and intensities, both in healthy controls and patients with obesity and associated metabolic diseases. Studies in humans are important, given that results could differ from those obtained in preclinical models, possibly aiding in the identification of the most viable therapeutic targets.

Altogether, exercise-factors are novel molecules that may, in the future, mark pathways towards new diagnostic and prognostic markers, as well as probable therapeutic strategies in obesity, especially considering that their basal levels, modulation by exercise, and functions could be blunted in this context, at least initially. Impairment of the modulation by exercise of these exercise-inducible factors or resistance to their actions in target tissues could perhaps be overcome by repeated and uninterrupted exercise training regimes, which we recognize may not always be a suitable option for patients with morbid obesity. Therefore, consideration of a pharmacologic alternative mimicking the effect of exercise-induced organokines could be an option in the future. Clinical trials have demonstrated controversial results, as exemplified by the successful results with an FGF21 analog [286] and the less-than-expected results obtained with exogenous leptin in non-hereditary obesity (in the absence of concomitant dietary energy restriction) [288]. Given the crosstalk among the organokines, a potential candidate for clinical trials would be an organokine that not only exerts direct effects on target tissues but also mediates additional favorable interactions with other humoral mediators. Irisin, for example, is a promising candidate which has been shown to mediate pleiotropic metabolic benefits and to possess anti-inflammatory properties in preclinical models. Nonetheless, many aspects of these molecules in general remain understudied or controversial in humans (e.g., circulating half-life, protein binding in circulation, degradation sites, receptors, elicited signaling pathways, effects in all target tissues), and thus their inclusion in clinical trials is still a long way off. Moreover, given the pleiotropic nature of these factors and their simultaneous regulation by exercise, exercise should be considered as an important influential means in humans to enhance metabolic health independently of weight loss, while also enhancing nutritional and psychosocial well-being, in the prevention and treatment of obesity. Specific indications for pharmacologic and surgical options for the treatment of obesity have been described and should be considered when recommended. Nevertheless, we emphasize the importance of exercise interventions for a healthy lifestyle and to prevent and aid in the treatment of obesity and its metabolic complications.

## Figures and Tables

**Table 1:** Myokines summary. Exercise modality evaluated in clinical studies is aerobic and conducted in adult populations unless specified otherwise. ↓: Decreased; ↑: Increased; ↔: Unchanged; ?: Controversial; *: Possible mechanism (based on indirect evidence); -: Data not available; °: Depending on the specific context, may be either pro- or anti-inflammatory; +: Indicates reviews or meta-analyses; AE: Acute exercise; CE: Chronic exercise; Ob: Obesity; Ow: Overweight; T2DM: Type 2 diabetes mellitus. Other abbreviations are given throughout the text.

Myokine & Status in Metabolic Disease	Status with Exercise (Clinical Studies)	Known and Potential Endogenous Inhibitors and Stimuli	Modulation of Other Mediators	Effects on Metabolism
**Irisin** Ob: ↑ [39]+; T2DM: ↓ [39]+	AE: ↑ (*aerobic or resistance*) [40]+ CE: ↓? *(aerobic or resistance)* [41]+	↑ (via PGC-1α): cold-induced shivering [42], ↑AMP:ATP ratio (via AMPK-PGC-1α pathway), FGF21* [43], and METRNL (via ↑PGC-1α4)* [44] **↓**: proinflammatory cytokines [45] (possibly via NF-kB-mediated inhibition of PGC-1α)* [46], glucolipotoxicity [47,48], myostatin [49], and selenoprotein P (via inhibition of ROS-AMPK-PGC-1α pathway* [50])	↑: IL-10 by Mφ [49], adiponectin [51], BDNF by hypothalamus [52] ↓: TNF-α, IL-1β, IL-6, and MCP-1 by Mφ and adipocytes [51,53]; ? leptin by adipocytes [54,55]; and myostatin by myocytes [44]	**AT/Immune**: ↑WAT browning, thermogenesis, and energy expenditure [56,57]; ↑WAT lipolysis and ↓lipid accumulation [58]; anti-inflammatory [49,51,53,59]; antioxidant [60,61] **SkM**: ↑glucose and FA uptake and oxidation [62,63]; ↓glycogenolysis and ↑glycogenesis [63]; ↑mitochondrial biogenesis and thermogenesis [64] **Liver:** ↓lipid accumulation [65] **CNS/Others:** ↓β-cell apoptosis [66]; ?↑appetite control and energy expenditure (via BDNF and other anorexigenic peptides) [52,67,68]
**IL-6°** Ob and T2DM: ↑ [69]+	AE: ↑ (Healthy [70]+) CE: ↓ (*basal levels*) (Healthy and Ob [70]+)	↑ (via MAPK/JNK/AP-1): OCN [71], oxidative stress [70]+ ↓: glucose intake during exercise [70]+	↑: IL-10, IL-1Ra, cortisol [72], GLP-1 [73] (indirectly ↑insulin), and OCN [71] ↓: TNF-α [9]+ and IL-1β [74]	**AT/Immune:** ↑lipolysis [75] and whole-body FAO [76,77] → ↑visceral weight loss [78]; ↑BAT activation [16]+ and WAT browning [79]+**;** anti-inflammatory (when released by SkM [9]+); ↑M2 Mφ activation [80] **SkM:** ↑insulin sensitivity and glucose disposal [76] (also via ↑GLP-1) [73] **Liver:** ↑glucose production [69]+ **CNS**: suppression of feeding (directly [7,81,82] and via GLP-1 [83])
**IL-15** Ob: ↓ [84]+; T2DM: ?↓ [84]+	AE: ? ↑↔, (Healthy, Ob, and T2DM [84]+) CE: ? ↑(Healthy [85]); ? ↔ (*Ob and T2DM* [84]+; *possibly due to increased sIL-15Ra*)	-	↑: adiponectin [86] ↓: TNF-α* [26]+	**AT/Immune:** antioxidant [87], anti-inflammatory [26]+, ↓lipid accumulation in adipocytes [88] (↑lipolysis [89], ↓lipogenesis [90]) **SkM:** ↑mitochondrial biogenesis → ↑FAO (↑PPARδ, PGC-1α/β, SIRT1) [84,91]; ↑glucose uptake, insulin sensitivity [84]+ **;** muscle hypertrophy [26]+
**METRNL** Ob: ↑ [92]; T2DM: ?↑ [92,93,94], ↓ [95,96]	AE: ↑ (Healthy, *combined aerobic and resistance; HIIT* [97,98] and Ow [99]) CE: ↑ (Healthy, *HIIT* [97])	↑ (via PGC-1α4 [98]): cold exposure (in WAT) ↓: elevated FFAs [100]	↑: IL-4 and IL-13 by Eos [98], irisin, and BAIBA (via ↑PGC-1α)* [100] ↓: TNF-α, MCP-1, and IL-6 [100]	**AT/Immune**: ↑eosinophils in AT (↑IL-4 and IL-13 → M2 polarization → ↑WAT browning) [12,98,101]; anti-inflammatory [100]; ↑insulin sensitivity [102] **SkM:** ↑insulin sensitivity [100]**;** ↑FAO (via AMPK and PPARδ) [100] and glucose uptake [103]
**BAIBA** Ow/Ob: ?↓ (trends only) [104,105]	AE: ? ↑ [106] ↔ [107] (Healthy) CE: ↑ [104] (Healthy)	↑: METRNL (via ↑PGC-1α)* [100,104]	↑: OCN by osteoblasts [108]; leptin by adipocytes [109] ↓: TNF-α and MCP-1 by adipocytes [110]	**AT/Immune**: ↑browning (↑UCP1, PRDM16, CIDEA) [104]; ↓inflammation (via AMPK → ↓NF-κB) and ↑insulin sensitivity [110]; ↓lipogenesis and adipogenesis [110] **SkM**: ↓inflammation and insulin resistance (via AMPK-PPARδ) [111]; ↑β-oxidation (via AMPK-PPARδ) [111] **Liver:** ↑β-oxidation (via PPAR-α) [104] and ↓lipogenesis [112,113] **CNS/others:** ↓hypothalamic inflammation [114]
**Myostatin** Ob: ↑ [115]+	AE: ↓ (Healthy and Ob; *aerobic or resistance*) [6,115]+ CE: ↓ (Healthy and Ob; *aerobic or resistance*) [6,115]+	↑: sedentarism, TNF-α [116] ↓: follistatin [117], irisin [44]	↑: TNF-α [116] ↓: irisin [49]	**AT:** facilitates body fat accumulation [118,119] (↓myostatin → ↑lipolysis and FAO in adipocytes, ↑WAT “browning” via irisin) [44] **SkM**: ↓SkM growth (↓satellite cell proliferation and differentiation, ↓protein accretion) [115]+

**Table 2:** Hepatokines summary. Exercise modality, evaluated in clinical studies, is aerobic and was conducted in adult populations, unless specified otherwise. ↓: Decreased; ↑: Increased; ↔: Unchanged; ?: Controversial; *: Possible mechanism (based on indirect evidence); -: Data not available; +: Indicates reviews or meta-analyses; AE: Acute exercise; CE: Chronic exercise; MS: Metabolic syndrome; NAFLD: Non-alcoholic fatty liver disease; Ob: Obesity; Ow: Overweight; T2DM: Type 2 diabetes mellitus. Other abbreviations are given throughout the text.

Hepatokine and Status in Metabolic Disease	Status with Exercise (Clinical Studies)	Known and Potential Endogenous Inhibitors and Stimuli	Modulation of Other Mediators	Effects on Metabolism
**Selenoprotein P** Ob, NAFLD, T2DM: ↑ [23]+	AE: ↔ [50,134] CE: ↔ [50]	↑: hepatic steatosis [23]+ ↓: adiponectin [135]	↓: adiponectin [136], IL-6 and PGC-1α dependent myokines irisin, METRNL, BAIBA (by decreasing ROS*) [50]	**SkM**: antioxidant [50]; deficiency facilitates SkM adaptation to exercise via LRP1 (↓antioxidant SeP → ↑ROS → ↑AMPK and PGC-1α → ↑mitochondrial biogenesis and metabolic adaptation to exercise) [50]; ↑insulin resistance (possibly indirect via ↓adiponectin) [23]+ **Liver:** ↑insulin resistance [23]+
**Fetuin A** Ob, MS, NAFLD, T2DM: ↑ [137]+	AE: ↔ (Healthy and Ow/Ob) [134] CE: ↓ (Healthy [138]+ and Ow [139,140]); ? ↓ [141,142,143] ↔ [138] + (Ob w/NAFLD or T2DM)	↑: excess glucose (via ERK1/2) [144] or FFAs (via NF-KB) [145]; hepatic steatosis [23]+ ↓: adiponectin (via AMPK) [146], irisin (by decreasing hepatic lipogenesis*) [147]	↑: proinflammatory cytokines TNF-α, IL-6 [148] by adipocytes and monocytes (via TLR-4) [149] ↓: adiponectin [148]	**AT/Immune:** ↑inflammation [140,150]; ↑insulin resistance; ↑FFA uptake and lipogenesis [151] **SkM:** ↑insulin resistance [23]+ **Liver:** ↑insulin resistance [23]+ **Others:** ↑β-cell lipotoxicity and apoptosis [152] → ↓insulin secretion
**FGF21** Ob, NAFLD and T2DM: ↑ [23,153,154]+	AE: ↑ (Healthy [155]); ↔ (Ob [156] and T2DM [157]) CE: ↑ (T2DM; *aerobic or resistance*) [143]	↑: glucagon-to-insulin ratio (in liver) [158,159]; FFAs (via PPAR-α in liver) [159,160,161]; T3 (in liver) [162]; cold exposure (in BAT) [154]+ ↓: insulin [158]; T3 (in AT) [162]	↑: catecholamines [163], glucocorticoids [164], irisin (via PGC-1α in adipocytes)* [43], adiponectin [165]	**AT/Immune:** ↑SNS-induced BAT energy expenditure [163,165]; ↑PGC-1α and WAT browning [43]; ↑WAT lipolysis [166] **Liver:** ↑hepatic FAO [160]; ↓lipogenesis (↓SREBP-1c) [167] → ↓hepatic steatosis; ↑insulin sensitivity (with adiponectin) [154] **CNS:** ↓sugary food intake [168]
**ANGPTL4** Ob: ↑ [23]+ MS and T2DM: ?↑ [169] ↓ [23]+	AE: ↑ (Healthy [6,170,171] and Ow [171]; *aerobic or resistance*) CE: ? ↔ [6,172] ↑ (*marathon run)* [173]	↑: glucagon-to-insulin ratio (in liver) [170]; catecholamines → FFAs (via PPARs in liver and SkM) [174]; cortisol [175]; TLR stimulation (in Mφ) [169] ↓: glucose load (↑insulin → ↓lipolysis → ↓FFAs) [174]	-	**AT:** ↑WAT lipolysis [176,177], ↓LPL activity [178], → ↓fat mass [23]+ **Others:** ↓pancreatic lipase [179]
**Follistatin** NAFLD, T2DM: ↑ [180]	AE: ↑ (Healthy [117,181,182] *HIIT, aerobic, or resistance);* ↔ (T2DM [157]) CE: -	↑: glucagon-to-insulin ratio [183] ↓: -	↓: myostatin [184] and glucagon [183]	**AT:** ↑WAT browning [185] **SkM:** ↑hypertrophy (↑satellite cell activation due to↓ myostatin) [184] **Others:** protection of β-cells from apoptosis [183]

**Table 3:** Osteokines and adipokines summary. Exercise modality, evaluated in clinical studies, is aerobic and was conducted in adult populations unless specified otherwise. ↓: Decreased; ↑: Increased; ↔: Unchanged; ?: Controversial; *: Possible mechanism (based on indirect evidence); -: Data not available; +: Indicates reviews or meta-analyses; AE: Acute exercise; CE: Chronic exercise; MS: Metabolic syndrome; Ob: Obesity; Ow: Overweight; T2DM: Type 2 diabetes mellitus. Other abbreviations are given throughout the text.

Osteokine/Adipokine Status in Metabolic Disease	Status with Exercise (Clinical Studies)	Known and Potential Endogenous Inhibitors and Stimuli	Modulation of Other Mediators	Effects on Metabolism
**Osteocalcin (OCN)** Ow, Ob: ↓ [202]+; MS, T2DM: ↓ [203]+	AE: ↑ (Ob; *HIIT* [214]) CE: -	↑: IL-6 (via ↑RANKL and ↓OPG resulting in osteoclast differentiation) [71]; insulin [210] ↓: age [71]	↑: IL-6 by SkM [71], adiponectin and IL-10 by adipocytes [211], and insulin by β-cells (via GPRC6A) [209] ↓: TNF-α by adipocytes [211]	**AT**: ↑PGC-1α and UCP-1 expression in BAT [212]; ↑insulin sensitivity [28]+ **SkM**: ↑FFA and glucose uptake and oxidation [71]; ↑PGC-1α and mitochondrial biogenesis [28]+; ↑insulin sensitivity [28]+ **Liver:** ↓hepatic inflammation and lipid accumulation [213,215]; ↑insulin sensitivity [28]+ **Others**: ↑insulin secretion [209]
**Leptin** Ob: ↑↑ [216]+	AE: ↔↓ (Healthy; *aerobic or resistance* [217]+); ↔ [218]+ ↓ [6,217]+ (Ow and Ob) CE: ↓ (Healthy [219]+, Ow, Ob, and T2DM [218,219,220]+; *aerobic, resistance, or combined)*	↑: fat mass [216]+, BAIBA [109] ↓: prolonged fasting, β-adrenergic signaling [221], ? irisin [54,55]	↑: TNF-α, IL-1β, IL-6 by monocytes [222,223]; IL-2 and IFN-γ by T-cells [216]+ ↓: IL-4 by T-cells [216]+, irisin-induced WAT browning [224], and insulin [69]+	**CNS:** ↓food intake and ↑global energy expenditure (actions in hypothalamus) [225]+ **AT/Immune:** proinflammatory [222,223,226]+ **SkM:** ↑glucose and FFA uptake and oxidation [217]+ **Liver:** ↑FAO → ↓lipid accumulation [217]+
**Adiponectin** Ob: ↓ [216]+	AE: ↑ (Healthy [217]+; *aerobic or resistance*) ↔ (Ow and Ob [218]+) CE: ↑ (Ow, Ob [218,220]+) ? ↔↑ (T2DM [227]+)	↑: β-adrenergic signaling [69], IL-15 [86], FGF21 [165], irisin [51], OCN [211] ↓: hypoxia and oxidative stress [228], insulin [229], TNF-α [229], fetuin A [148], selenoprotein P [136]	↑: irisin (via AMPK-PGC-1α)* [230]+, IL-10 by Mφ [230]+ ↓:TNF-α, MCP-1, IL-6 by Mφ [230]+, fetuin A [146]	**CNS:** ↑food intake [230]+, ↓hypothalamic inflammation [231] **AT/Immune:** anti-inflammatory (↓differentiation of monocytes from myeloid progenitor cells, ↑M2 Mφ polarization, ↓TLR4 expression) [230]+ **SkM:** ↑FAO (via AMPK); ↑insulin sensitivity [230]+ **Liver**: ↑FAO (via AMPK), ↓lipogenesis; ↓gluconeogenesis, ↑insulin sensitivity [230]+
**Resistin** Ob and TD2M: ↑ [232]+	AE: ↔ (Ow [6]+) CE: ? (T2DM ↔ [227]+ ↓ [233]+; *aerobic or resistance*)	↑: TNF-α, IL-1β, IL-6 [232]+	↑: TNF-α, IL-6 [234]+, MCP-1 [232]+	**Systemic:** ↑insulin resistance [232]+; proinflammatory (↑TLR-4 activation) [234,235]+

**Table 4:** Immune cytokines summary. Exercise modality, evaluated in clinical studies, is aerobic and was conducted in adult populations unless otherwise specified. ↓: Decreased; ↑: Increased; ↔: Unchanged; ?: Controversial; *: Possible mechanism (based on indirect evidence); -: Data not available; °: Depending on the specific context, may be either pro- or anti-inflammatory; +: Indicates reviews or meta-analyses; AE: Acute exercise; CE: Chronic exercise; Ob: Obesity; Ow: Overweight; T2DM: Type 2 diabetes mellitus. Other abbreviations are given throughout the text.

Cytokine Status in Metabolic Disease	Status with Exercise (Clinical Studies)	Known and Potential Endogenous Inhibitors and Stimuli	Modulation of Other Mediators	Effects on Metabolism
**TNF-α** Ob and T2DM: ↑[239]+	AE: ↔↓ [6,8]+ ↑(*if intensive enough to cause SkM damage* [6]+) CE: ↓ (Healthy [249]+), ↓ (T2DM; *combined aerobic and resistance* [253]+)	↑: IFN-γ [216]+; FFAs (via TLR4-NF-KB) [239]+; leptin [222,223]; oxidative stress [238]+ ↓: IL-6 [74], IL-10 [8]+; adiponectin [230]+; irisin [51,53,60]	↑: leptin [254], resistin [255] ↓: FGF21 action in AT (via ↓β-klotho) [256], adiponectin [229]	**Systemic:** proinflammatory [216]+, ↑insulin resistance [20]+ **AT**: ↓UCP1 expression in BAT and WAT (↓energy expenditure) [257]
**IL-1β** Ob and T2DM: ↑ [239]+	AE: ↔↓ [8] + [238]+ CE: ↓ (T2DM; *aerobic, resistance, and combined* [253]+)	↑: oxidative stress [238]+; DAMPs (via NLRs-NALP3 inflammasome) [239]+ ↓: IL-6 [74]; IL-1Ra [8]+	↑: TNF-α, IL-1β, IL-6 [14]+	**Systemic:** proinflammatory, ↑insulin resistance [239]+ **AT: ↓**UCP1 expression in BAT and WAT (↓energy expenditure**)** [258] **Others:** pancreatic β-cell damage [8]+
**MCP-1** Ob: ↑ [216]+	AE: ↑ (Healthy, Ob, T2DM; *aerobic or resistance* [6]+) CE: ? ↔↓ (Healthy and Ob; *resistance* [6]+); ↓ (Ob; *combined aerobic and resistance* [259])	↑: adipocyte hypertrophy, hypoxia, mechanical stress, FFAs, DAMPs, proinflammatory cytokines (via NF-KB signaling) [239]+ ↓: irisin [51,53]; adiponectin [230]+	-	**AT/Immune: ↑**monocyte infiltration in WAT and M1 Mφ polarization [239]+
**IL-4**	AE: - CE: ↔ (Healthy [249]+); ↑(Ow adolescents [260]; T2DM, *aerobic, resistance, and combined* [253]+)	↑: IL-13 [12]+ ↓: leptin [216]+	↑: IL-10 [248]+, IL-1Ra [261]+ ↓: TNF-α, IL-1, IL-6 [261]+	**AT/Immune:** ↑M2 polarization → ↑insulin sensitivity; ↑Catecholamine production ? [12,101]+; (↑thermogenic gene expression, FA mobilization, energy expenditure) [262]
**IL-6°** Ob and T2DM: **↑** [69]+	AE: produced by SkM (see “myokines” above) CE (immune/adipose-derived): ↓ (Healthy [249]+ and T2DM; *aerobic, resistance, and combined* [253]+)	↑: adipocyte lipolysis, proinflammatory cytokines [69]+; leptin [216]+ ↓: adiponectin [230]+	↑: TNF-α and IL-1β [80]	**Systemic**: ↑insulin resistance (when released by activated immune cells) [122]+
**IL-10** Ob: ↓ [9]+	AE: ↑ (Healthy; *aerobic or HIIT, duration-dependent* [250]+) CE: ↑ (T2DM; *aerobic, resistance, and combined* [253]+)	↑: IL-6 [74], adiponectin [230]+	↓: TNF-α and IL-1β [9,238]+; IL-6 and IL-8 [261]+	**Systemic:** anti-inflammatory (↓IFN-γ by Th1, ↓ TNF-α by M1 Mφ, ↑IL-10 by Tregs) [14]+; ↑insulin sensitivity [9]+
**IL-13**	AE: - CE: ↑ (Ow adolescents; HIIT [260])	↑: IL-33 [12]+	↑: IL-10 [12]+ ↓: TNF-α, IL-1, IL-8 [261]+	**AT/Immune:** ↑M2 polarization (↑insulin sensitivity, ↑catecholamine production ?) [12,101]+
**IL-33** Ob: ↑ [263]+ (*impaired action)*	AE: - CE: ↑ (T2DM, *combined aerobic and resistance*) [264]	-	↑: IL-4 and IL-13 (indirect, by increasing recruitment and activation of Eos) [12]+; IL-10 (T-reg expansion) [263]+	**AT/Immune:** anti-inflammatory (maintenance of ILC2 cells → ↑IL-5 and IL-13 → ↑IL-4 and IL-13 production by Eos → M2 polarization and T-regs → ↑IL-10 → ↑WAT browning, insulin sensitivity) [12]+

**Table 5:** Crosstalk between exercise-inducible factors. ↔: Denotes a feedback loop. -: No data available. *: Indicates a possible, unconfirmed mechanism based on the activated signaling pathway.

	Myokines	Hepatokines	Adipokines	Immune Cytokines	Osteokines	Other Hormones
**Myokines**	myostatin ↔ irisin [44,49] METRNL → ↑irisin, BAIBA* [92,100]	FGF21 → ↑irisin* [43] follistatin → ↓myostatin [184] SeP → ↓adiponectin [136], ↓irisin, BAIBA, METRNL (via ↓PGC-1α due to ↓OS)* [50]	irisin → ↑adiponectin [51], ?↓leptin [54,55] IL-15 → ↑adiponectin [86] BAIBA → ↑leptin [109]	irisin → ↓TNF-α, IL-1β, IL-6, MCP-1, ↑IL-10 [49,51,53] IL-6 → ↓TNF-α and IL-1β [74] ↑IL-10 and IL-1Ra [72] BAIBA → ↓TNF-α and MCP-1[110] METRNL → ↑IL-4 and IL-13[98] ↓TNF-α, MCP-1, IL-6 [100]	↑OCN ↔ ↑IL-6 [71] BAIBA → ↑OCN [108]	IL-6 → ↑GLP-1 [73], cortisol [72] lactate → ↓ghrelin [247]
**Hepatokines**		↓fetuin A → ↑FGF21, follistatin, ANGPTL4 (by decreasing hepatic insulin resistance)* [23]	↓adiponectin [148] ↔ ↓fetuin A [146], SeP [135] FGF21 → ↑adiponectin [287]	TNF-α → ↓FGF-21 (induces resistance via ↓β-klotho) [256] fetuin A → ↑TNF-α, IL-6 [148]	-	FGF21 → ↑cortisol [164] glucagon → ↑FGF21 [158,159], follistatin [183], ANGPTL4 [170], cortisol → ↑ANGPTL4 [175]
**Adipokines**			-	leptin → ↑TNF-α, IL-1β, IL-6 [222,223] adiponectin → ↓ TNF-α, IL-6, ↑IL-10 [230] resistin → ↑TNF-α, IL-6 [234] TNF-α → ↓adiponectin [229], ↑resistin [255] and leptin [254]	OCN → ↑adiponectin [211]	adiponectin → ↑insulin sensitivity [230] insulin → ↑leptin [19] leptin → ↓insulin [69] catecholamines → ↑adiponectin, ↓leptin [69]
**Immune cytokines**				IL-10 → ↓TNF-α, IL-1β [248] IL-1Ra → ↓ IL-1β [8] IL-33 → ↑IL-4 and IL-13 → ↑IL-10 [12]	OCN → ↑IL-10, ↓TNF-α [211]	-
**Osteokines**					-	OCN → ↑insulin [209]
**Other hormones**						glucagon ↔ insulin catecholamines, cortisol, GH → ↓insulin (*antagonistic effect*)

**Table 6:** Summary of the role of the organokines in metabolism and energy balance. Details and references in text and in Table 1, Table 2, Table 3 and Table 4. ↑: Increased; ↓: Decreased; ?: Controversial or inconclusive; -: No effect or no data available.

Organokines	Central Energy Expenditure	Meta-Inflammation	Insulin Sensitivity	Active BAT	Role in Metabolic Pathway Regulation for Fat Redistribution and Loss
**Myokines**					
Irisin	↑ (via BDNF) ?	↓	↑ (also protects β-cells)	↑	↑lipolysis in WAT; ↓fat accumulation in WAT and liver; ↑glucose and FA oxidation in SkM
IL-6	↑ (also via GLP-1)	context-dependent	↑	↑	↑lipolysis in WAT; ↑glucose export by liver; ↑glucose and FA oxidation in SkM
IL-15	-	↓	↑	-	↑lipolysis and ↓lipogenesis in WAT; ↑FAO in SkM and hypertrophy (↑global EE)
METRNL	-	↓	↑	↑	↑glucose and FA oxidation in SkM
BAIBA	↑	↓	↑	↑	↓lipogenesis in WAT and liver; ↑FAO in SkM and liver
Myostatin	-	↑	↓	↓	↓lipolysis and FAO in WAT, SkM atrophy (↓global EE)
**Hepatokines**					
Selenoprotein P	-	-	↓	-	-
Fetuin A	-	↑	↓ (also detrimental for β-cells)	-	↑lipogenesis in WAT and liver
FGF21	↑	-	↑	↑	↑lipolysis in WAT; ↑FAO and ↓lipogenesis in liver
ANGPTL4	-	-	-	-	↑lipolysis and ↓lipid accretion in WAT; ↓dietary fat absorption
Follistatin	-	-	- (protects β-cells)	↑	↑ SkM hypertrophy (↑global EE)
**Osteokine**					
OCN	↑ (via insulin) ?	↓	↑ (also promotes secretion)	↑	↑glucose and FA oxidation in SkM; ↓lipid accumulation in liver
**Adipokines**					
Leptin	↑	↑	-	-	↑glucose and FA oxidation in SkM; ↑FAO in liver
Adiponectin	↓	↓	↑	↑	↑ FAO in SkM and liver; ↓lipogenesis in liver
Resistin	-	↑	↑	-	-

## Literature Search

We performed an exhaustive PubMed database search to find published articles focused on endocrine modulation by physical exercise, using as key words “physical activity”, “exercise”, “obesity”, “metabolic syndrome”, “diabetes”, “insulin resistance”, “inflammation”, “energy expenditure”, “browning”, “fat mass”, “visceral fat”, “organokines”, “myokines”, “hepatokines”, “adipokines”, “osteokines”, “cytokines”, and the specific organokines’ names (e.g., “irisin”, “IL-6”, “IL-15”, “METRNL”, “BAIBA”, “myostatin”, “selenoprotein P”, “fetuin A”, “FGF21”, “ANGPTL4”, “follistatin”, “osteocalcin”, “leptin”, “adiponectin”, and “resistin”), sorted by relevance and by date.

## Abbreviations

ADAM	A disintegrin and metalloproteinase
AMPK	AMP-activated protein kinase
AE	Acute exercise
ANGPTL4	Angiopoietin-like 4
AP-1	Activator protein-1
AT	Adipose tissue
BAIBA	Beta-aminoisobutyric acid
BAT	Brown or beige adipose tissue
BMR	Basal metabolic rate
CE	Chronic exercise
CIDEA	Cell-death inducing DNA fragmentation factor-like effector A
CNS	Central nervous system
DEXA	Dual-energy X-ray absorptiometry
EE	Energy expenditure
FAO	Fatty acid oxidation
FFA	Free fatty acid
FGF21	Fibroblast growth factor 21
FI	Food intake
FNDC5	Fibronectin type III domain-containing protein 5
GLP-1	Glucagon-like peptide-1
GLUT4	Glucose transporter type 4
HIIT	High-intensity interval training
HSL	Hormone sensitive lipase
IFN-γ	Interferon gamma
IL	Interleukin
IRS-1	Insulin receptor substrate 1
JNK	c-Jun N-terminal kinase
Mφ	Macrophage
MAPK	Mitogen-activated protein kinase
MCP-1	Monocyte chemoattractant protein-1
METRNL	Meteorin-like
MS	Metabolic syndrome
NAFLD	Non-alcoholic fatty liver disease
NF-κB	Nuclear factor kappa B
Ob	Obesity
OCN	Osteocalcin
OS	Oxidative stress
Ow	Overweight
PGC-1α	Peroxisome proliferator-activated receptor-gamma coactivator 1-alpha
PKC	Protein kinase C
PPAR	Peroxisome proliferator-activated receptor
PRDM16	PR domain containing 16
ROS	Reactive oxygen species
scAT	Subcutaneous adipose tissue
SeP	Selenoprotein P
SkM	Skeletal muscle
SNS	Sympathetic nervous system
T2DM	Type 2 diabetes mellitus
TG	Triglycerides
TLR4	Toll-like receptor 4
TNF-α	Tumor necrosis factor alpha
UCP1	Uncoupling protein 1
VAT	Visceral adipose tissue
WAT	White adipose tissue
